# Supplementary figures and images for: Humanized avian embryo models replicate an immune tumor environment for rapid immunotherapy studies
Source: EMBO Mol Med. 2026 Mar 19;18(4):1399–428. doi: 10.1038/s44321-026-00398-5 (PMC13083996; doi:10.1038/s44321-026-00398-5)

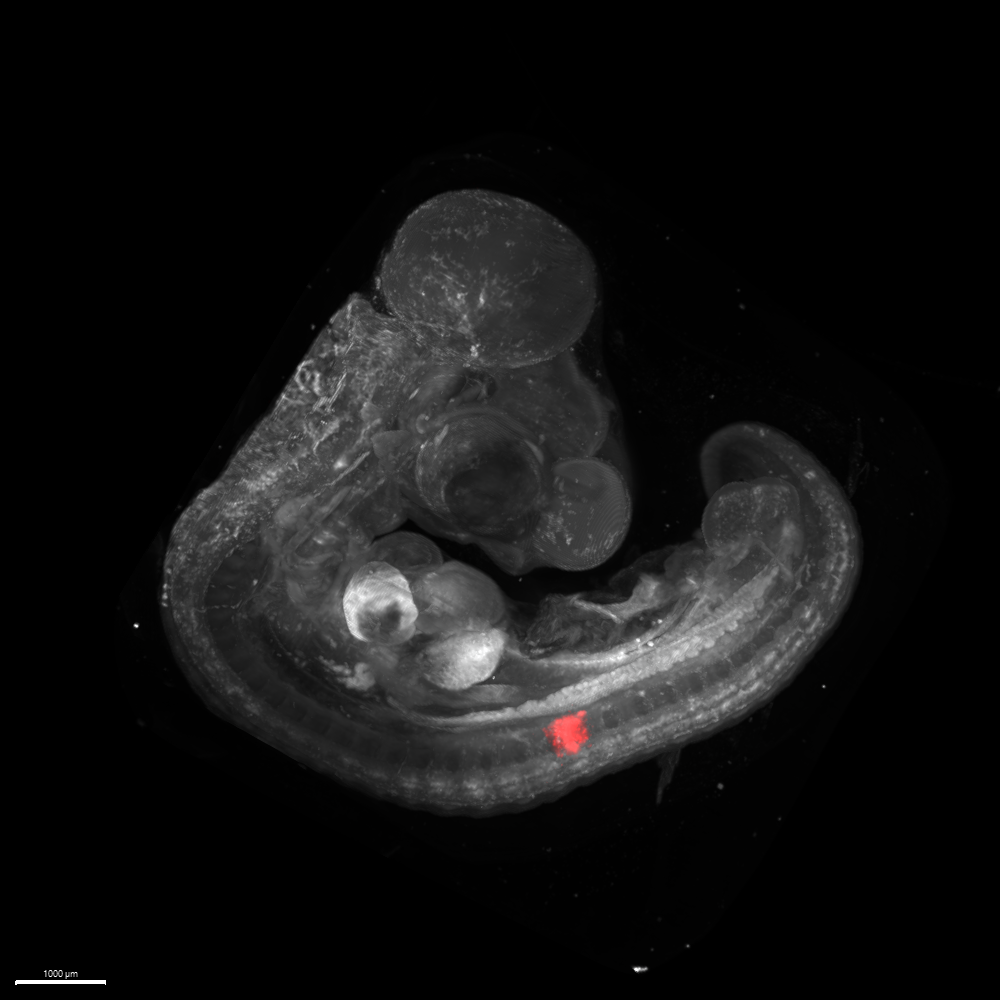

Supplement: Supplementary file 2 — Source data Fig. 1 [file 44321_2026_398_MOESM2_ESM.zip › 2025-21404-Figure1/1B/Entire embryo engrafted with Orange labelled-Hu-PBMC orange.tif]

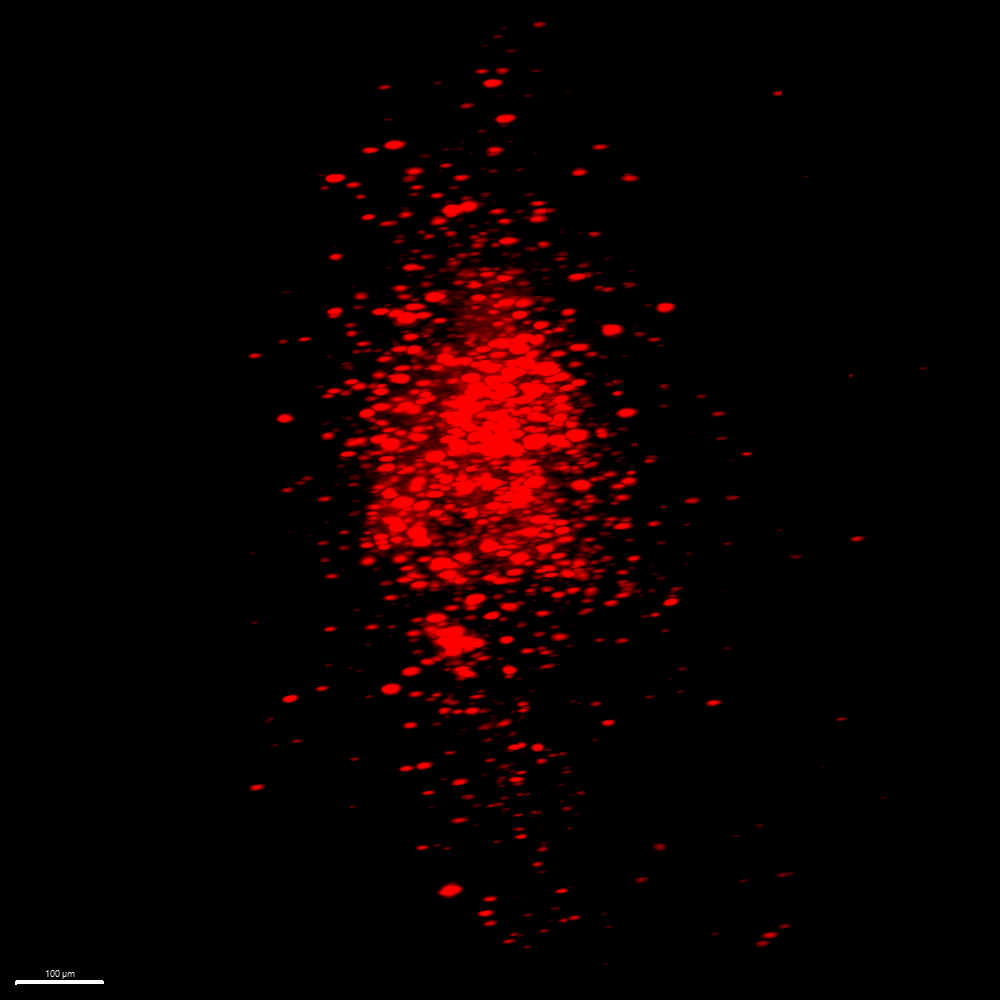

Supplement: Supplementary file 2 — Source data Fig. 1 [file 44321_2026_398_MOESM2_ESM.zip › 2025-21404-Figure1/1B/Fluorescent signal of Orange labelled-Hu-PBMC 1.tif]

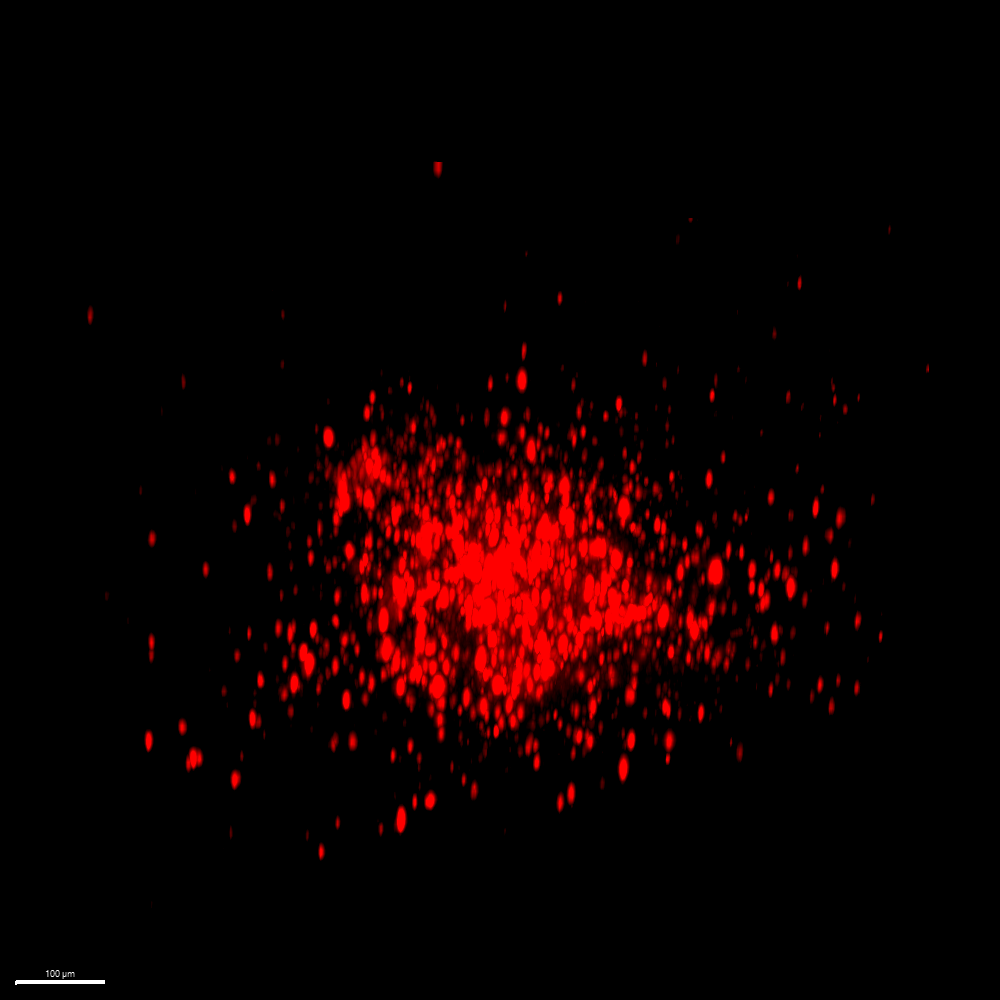

Supplement: Supplementary file 2 — Source data Fig. 1 [file 44321_2026_398_MOESM2_ESM.zip › 2025-21404-Figure1/1B/Fluorescent signal of Orange labelled-Hu-PBMC 2.tif]

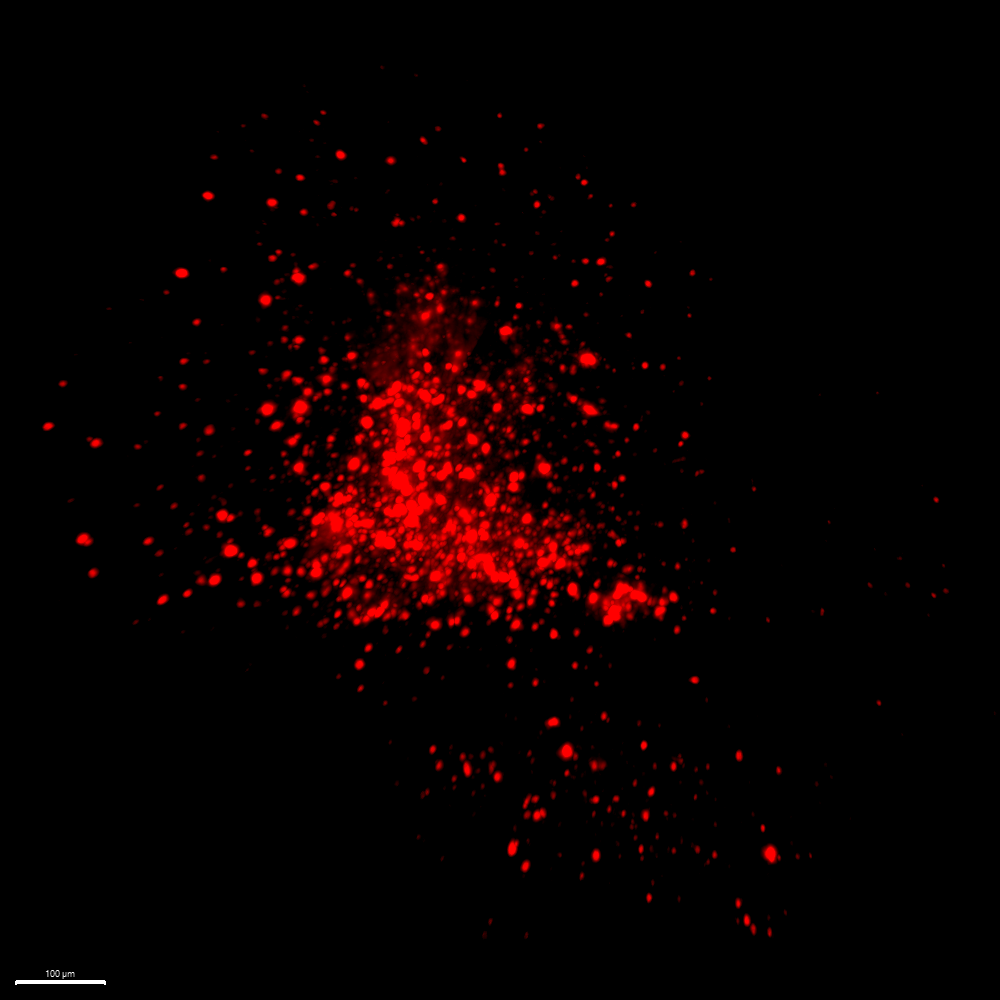

Supplement: Supplementary file 2 — Source data Fig. 1 [file 44321_2026_398_MOESM2_ESM.zip › 2025-21404-Figure1/1B/Fluorescent signal of Orange labelled-Hu-PBMC 3.tif]

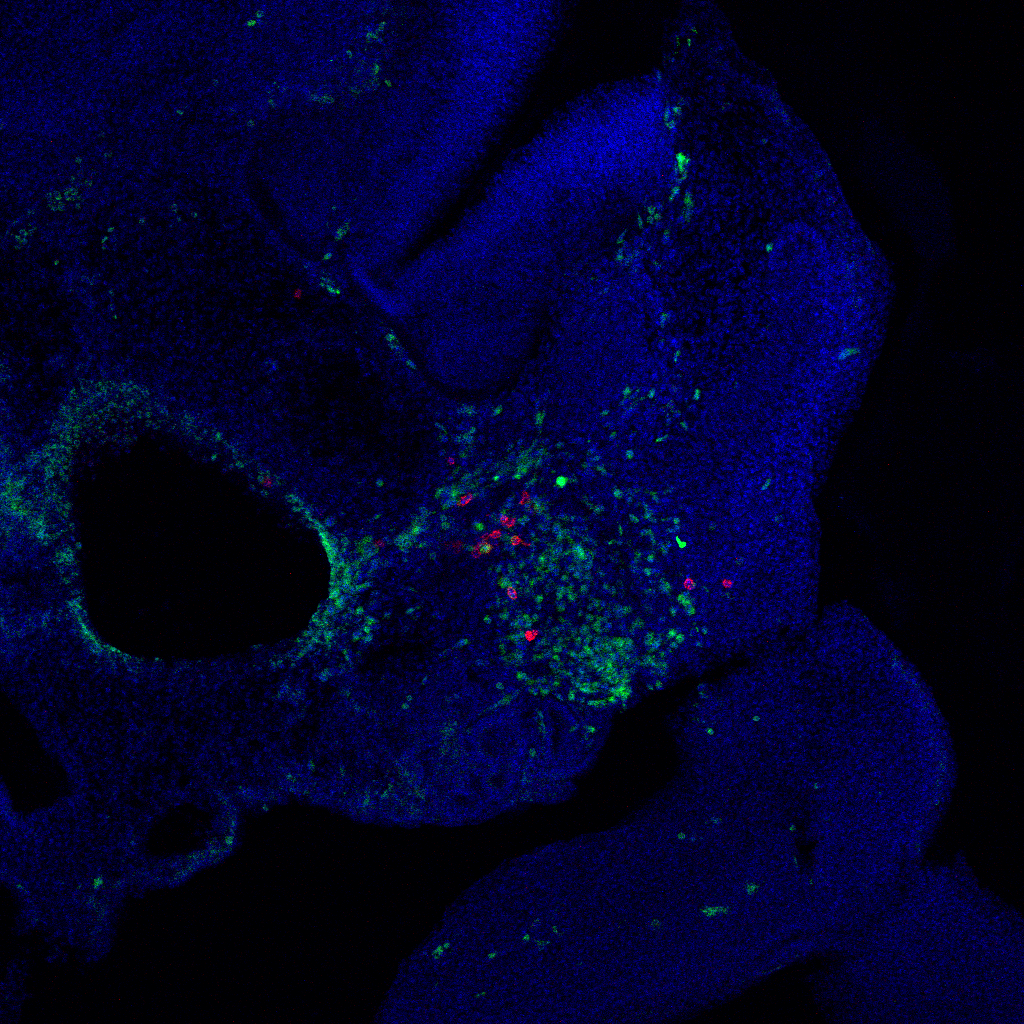

Supplement: Supplementary file 3 — Source data Fig. 2 [file 44321_2026_398_MOESM3_ESM.zip › 2025-21404-Figure2/2F/Embryo section x10 blue nucleus-green tumor cells-red Hu-CD45.tif]

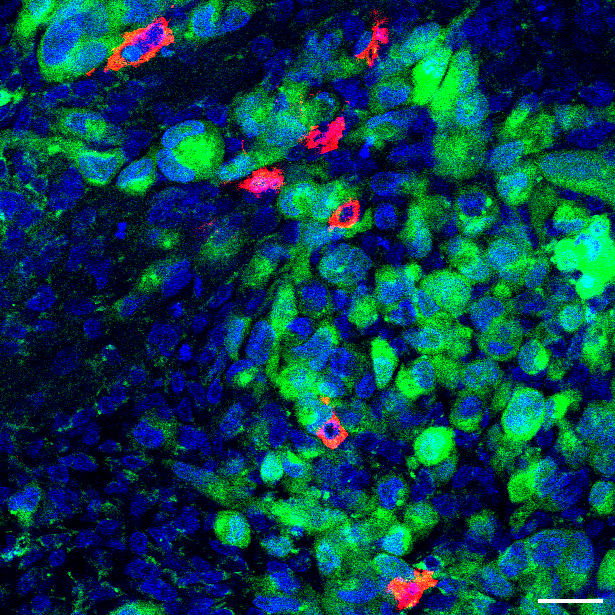

Supplement: Supplementary file 3 — Source data Fig. 2 [file 44321_2026_398_MOESM3_ESM.zip › 2025-21404-Figure2/2F/Embryo section x40 blue nucleus-green tumor cells-red Hu-CD45.png]

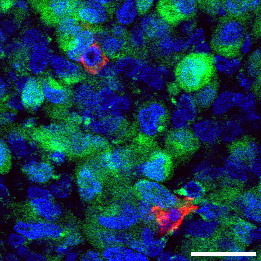

Supplement: Supplementary file 3 — Source data Fig. 2 [file 44321_2026_398_MOESM3_ESM.zip › 2025-21404-Figure2/2F/Embryo section-Zoom1- blue nucleus-green tumor cells-red Hu-CD45.png]

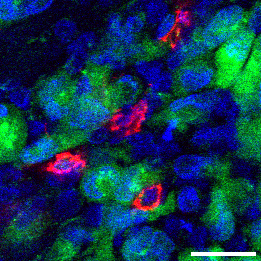

Supplement: Supplementary file 3 — Source data Fig. 2 [file 44321_2026_398_MOESM3_ESM.zip › 2025-21404-Figure2/2F/Embryo section-Zoom2- blue nucleus-green tumor cells-red Hu-CD45.png.png]

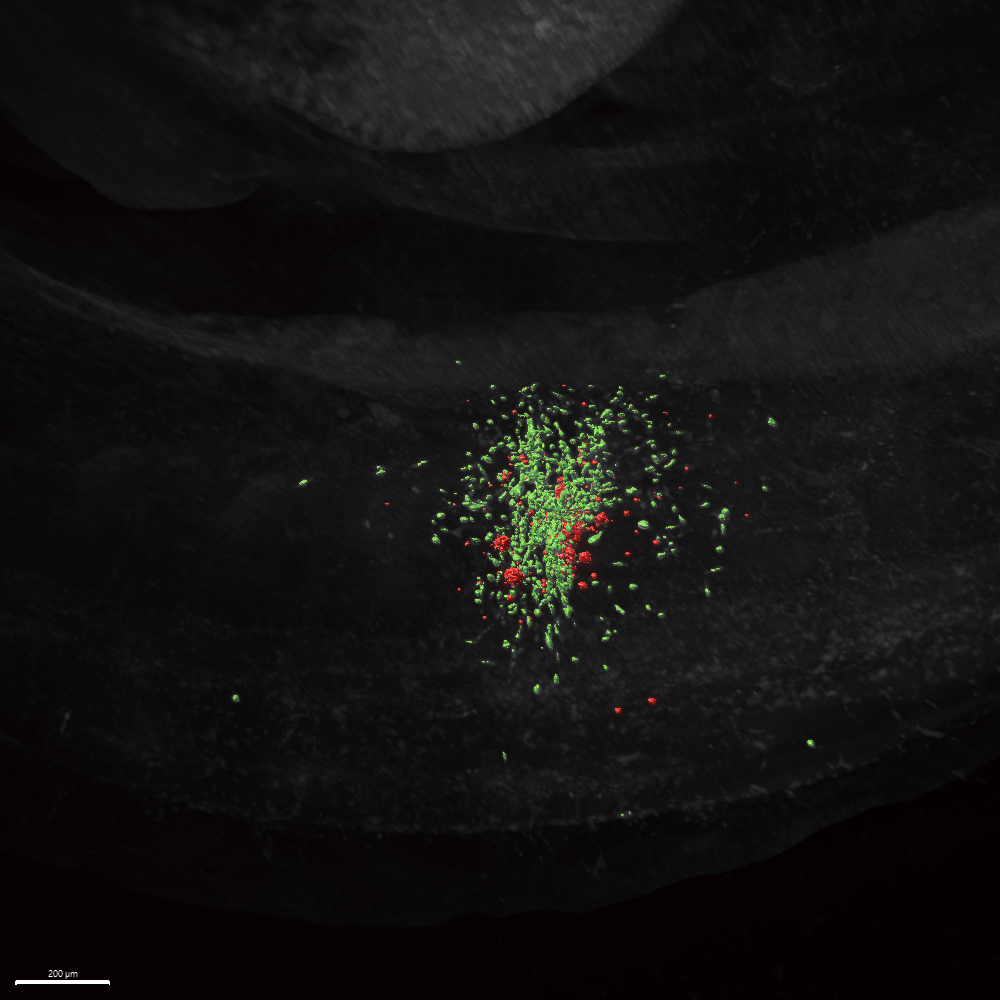

Supplement: Supplementary file 4 — Source data Fig. 3 [file 44321_2026_398_MOESM4_ESM.zip › 2025-21404-Figure3/3C/Co-grafted Hu PBMC orange- tumor cell line MDAMB 231 CFSE.tif]

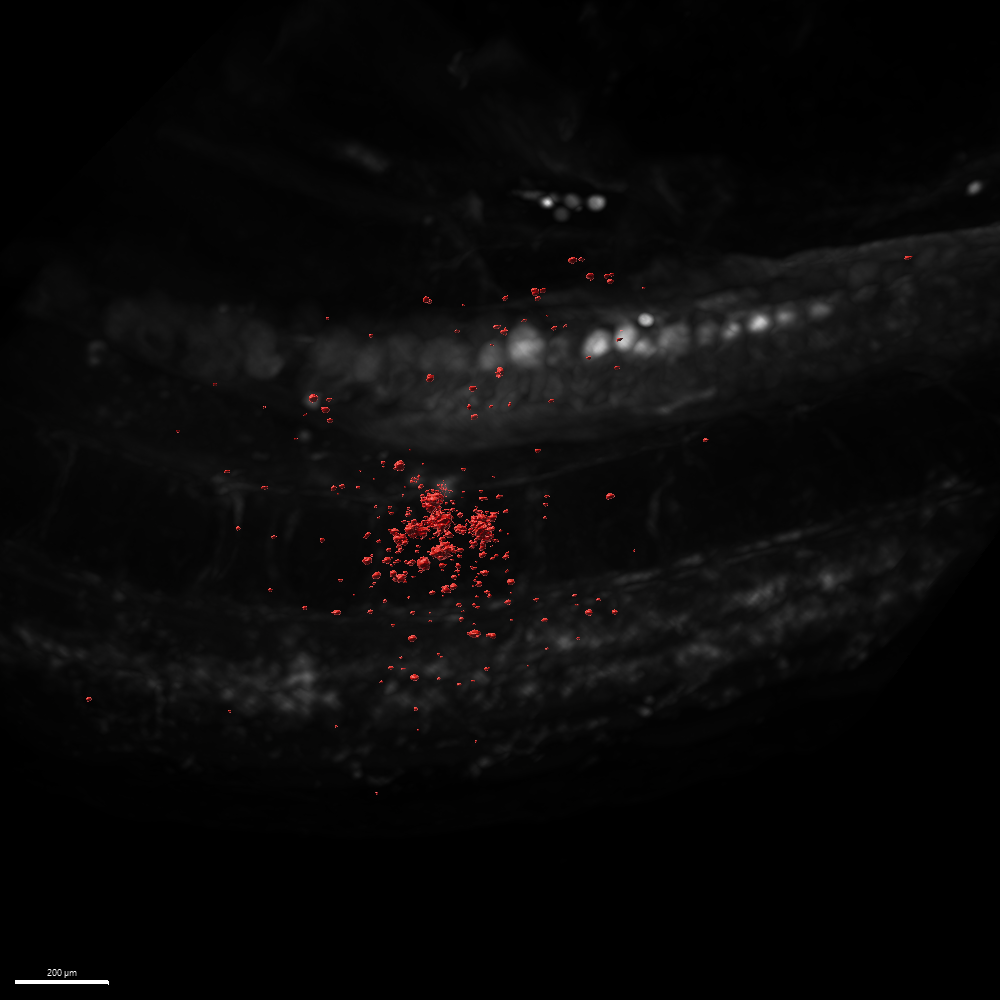

Supplement: Supplementary file 4 — Source data Fig. 3 [file 44321_2026_398_MOESM4_ESM.zip › 2025-21404-Figure3/3C/Grafted Hu PBMC orange.tif]

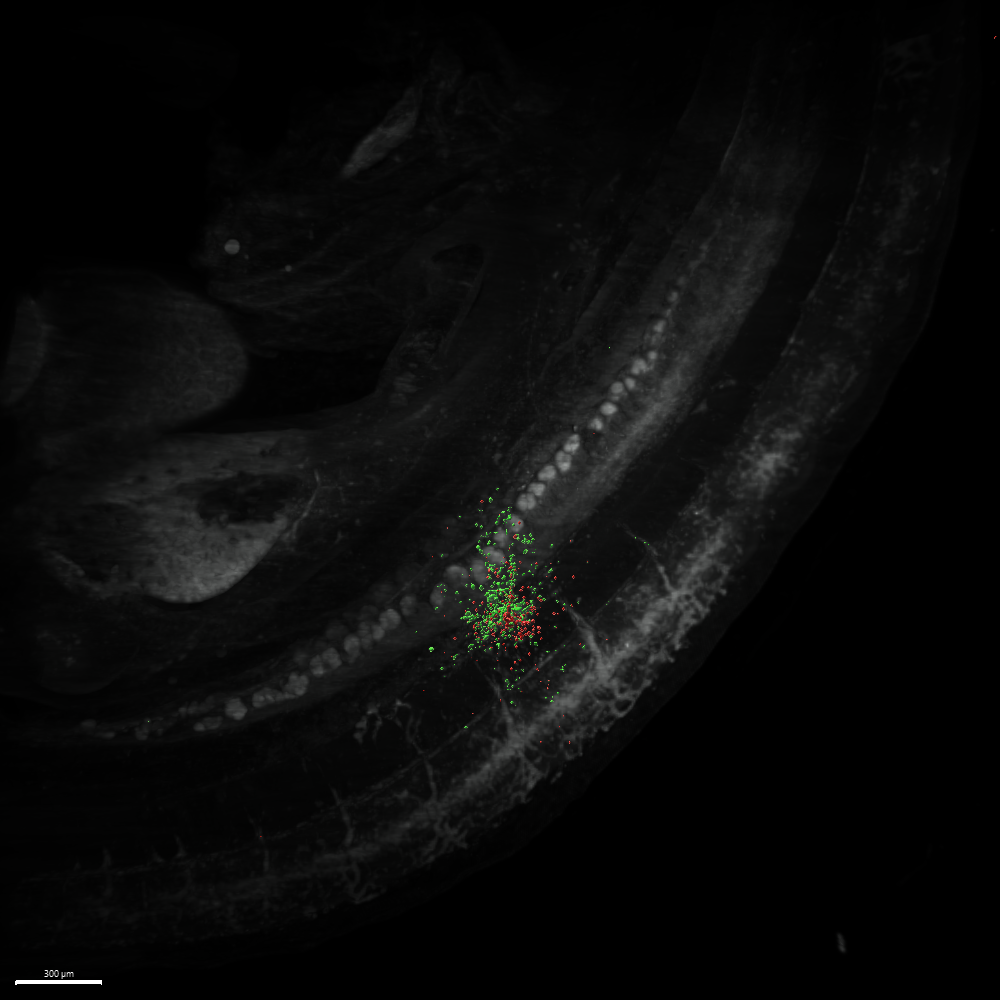

Supplement: Supplementary file 4 — Source data Fig. 3 [file 44321_2026_398_MOESM4_ESM.zip › 2025-21404-Figure3/3D/10-58-05_e5 mix ex567_blaze_c00_xyz-table z0000.ome_2024-11-27T10-52-44.532.tif]

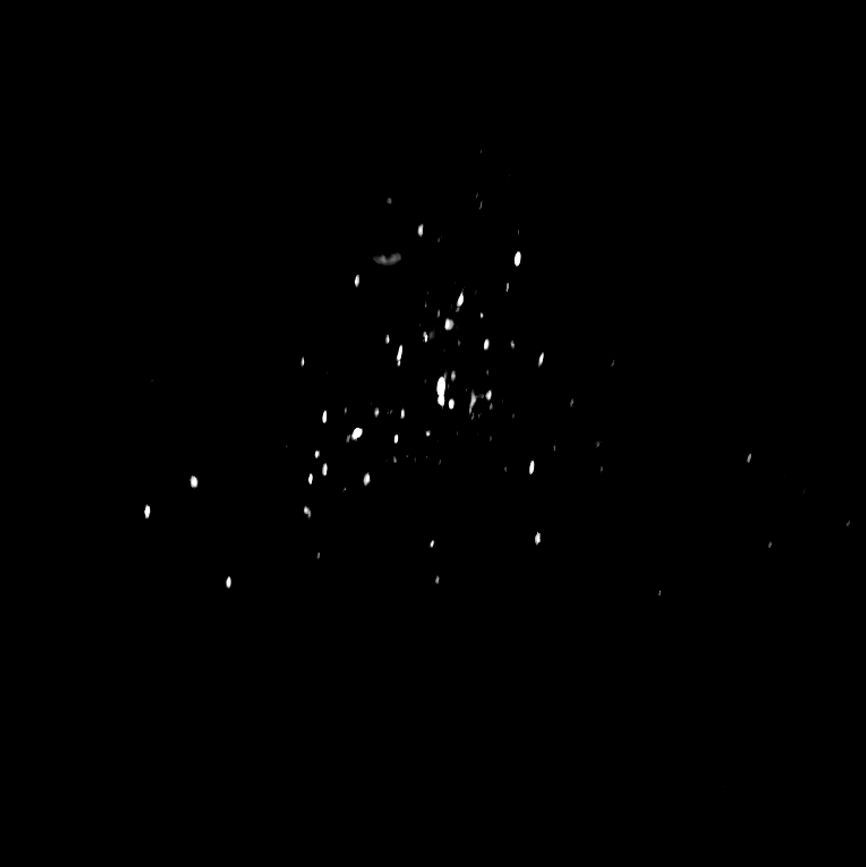

Supplement: Supplementary file 4 — Source data Fig. 3 [file 44321_2026_398_MOESM4_ESM.zip › 2025-21404-Figure3/3D/10-58-05_e5 mix ex567_blaze_colocalization.tif]

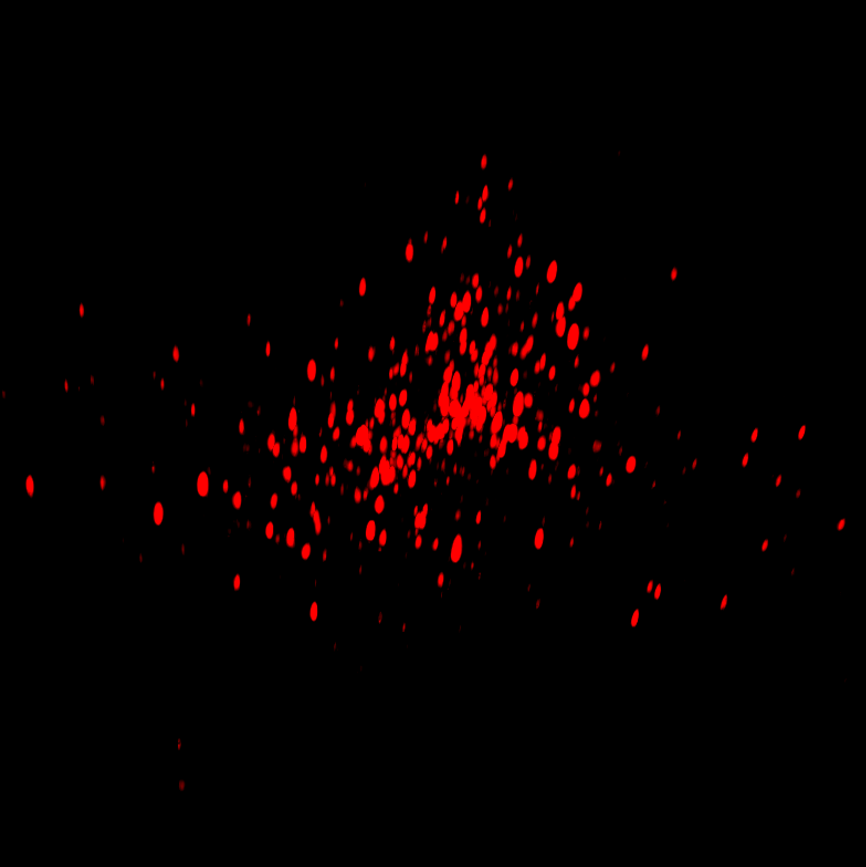

Supplement: Supplementary file 4 — Source data Fig. 3 [file 44321_2026_398_MOESM4_ESM.zip › 2025-21404-Figure3/3D/10-58-05_e5 mix ex567_blaze_huPBMC.tif]

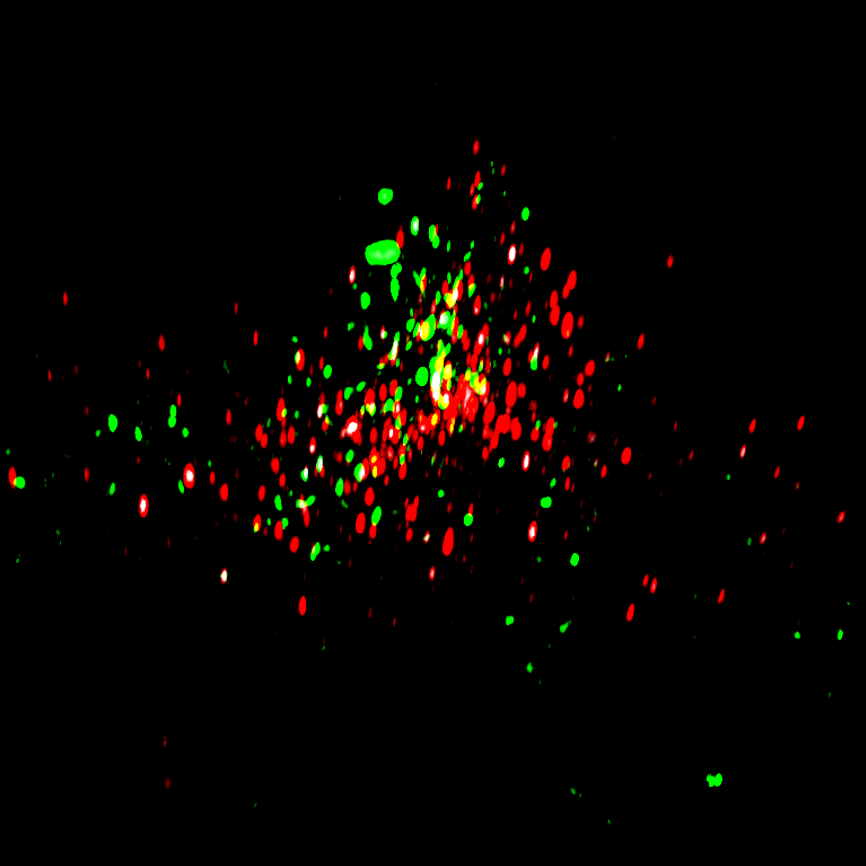

Supplement: Supplementary file 4 — Source data Fig. 3 [file 44321_2026_398_MOESM4_ESM.zip › 2025-21404-Figure3/3D/10-58-05_e5 mix ex567_blaze_MDAMB 231 and huPBMC.tif]

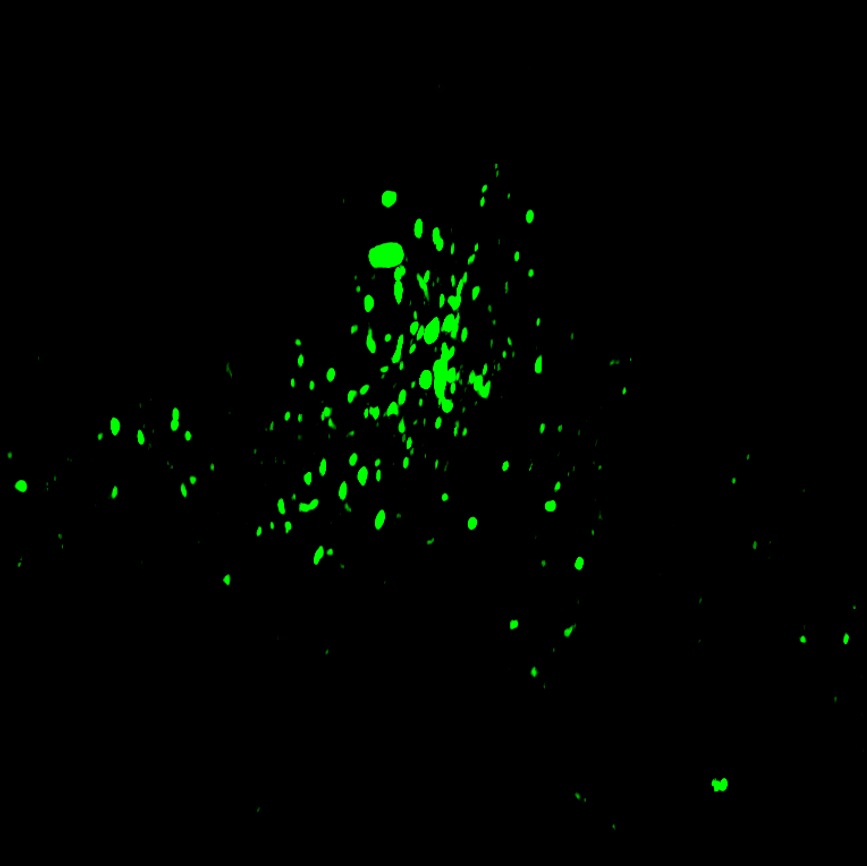

Supplement: Supplementary file 4 — Source data Fig. 3 [file 44321_2026_398_MOESM4_ESM.zip › 2025-21404-Figure3/3D/10-58-05_e5 mix ex567_blaze_MDAMB 231.tif]

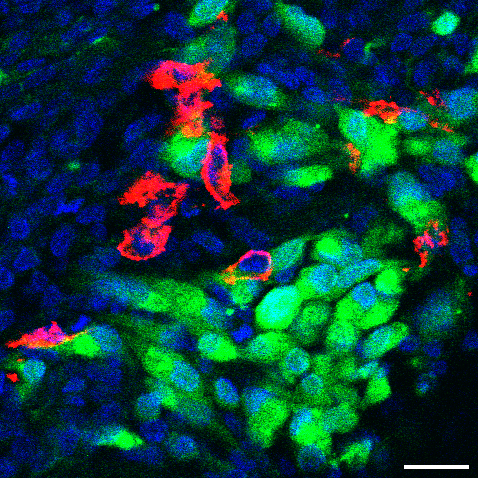

Supplement: Supplementary file 4 — Source data Fig. 3 [file 44321_2026_398_MOESM4_ESM.zip › 2025-21404-Figure3/3F/Co-engraftemnt- blue nucleus-green tumor cells-red Hu-CD45- 1.png]

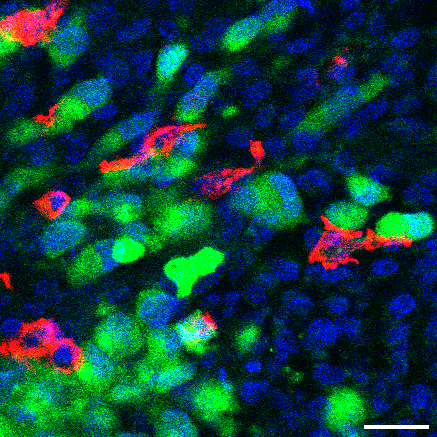

Supplement: Supplementary file 4 — Source data Fig. 3 [file 44321_2026_398_MOESM4_ESM.zip › 2025-21404-Figure3/3F/Co-engraftemnt- blue nucleus-green tumor cells-red Hu-CD45- 2.png]

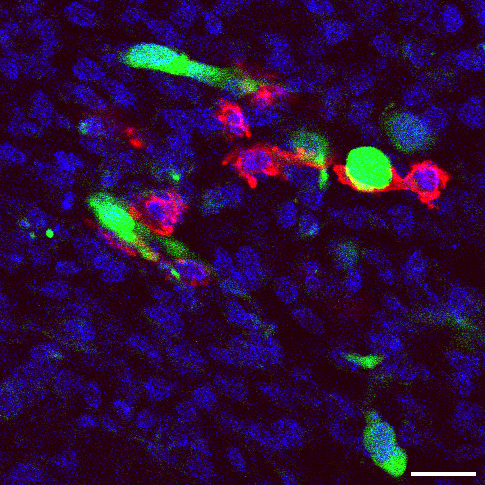

Supplement: Supplementary file 4 — Source data Fig. 3 [file 44321_2026_398_MOESM4_ESM.zip › 2025-21404-Figure3/3F/Co-engraftemnt- blue nucleus-green tumor cells-red Hu-CD45- 3.png]

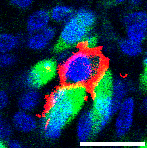

Supplement: Supplementary file 4 — Source data Fig. 3 [file 44321_2026_398_MOESM4_ESM.zip › 2025-21404-Figure3/3F/Co-engraftemnt- blue nucleus-green tumor cells-red Hu-CD45- 4 zoom.png]

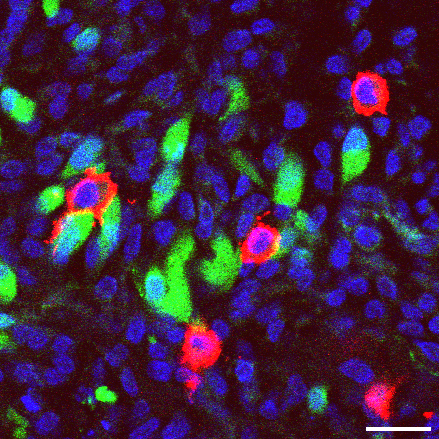

Supplement: Supplementary file 4 — Source data Fig. 3 [file 44321_2026_398_MOESM4_ESM.zip › 2025-21404-Figure3/3F/Co-engraftemnt- blue nucleus-green tumor cells-red Hu-CD45- 4.png]

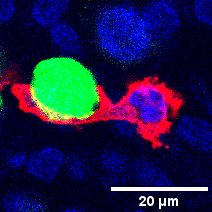

Supplement: Supplementary file 4 — Source data Fig. 3 [file 44321_2026_398_MOESM4_ESM.zip › 2025-21404-Figure3/3F/Co-engraftemnt- blue nucleus-green tumor cells-red Hu-CD45-3 zoom.png]

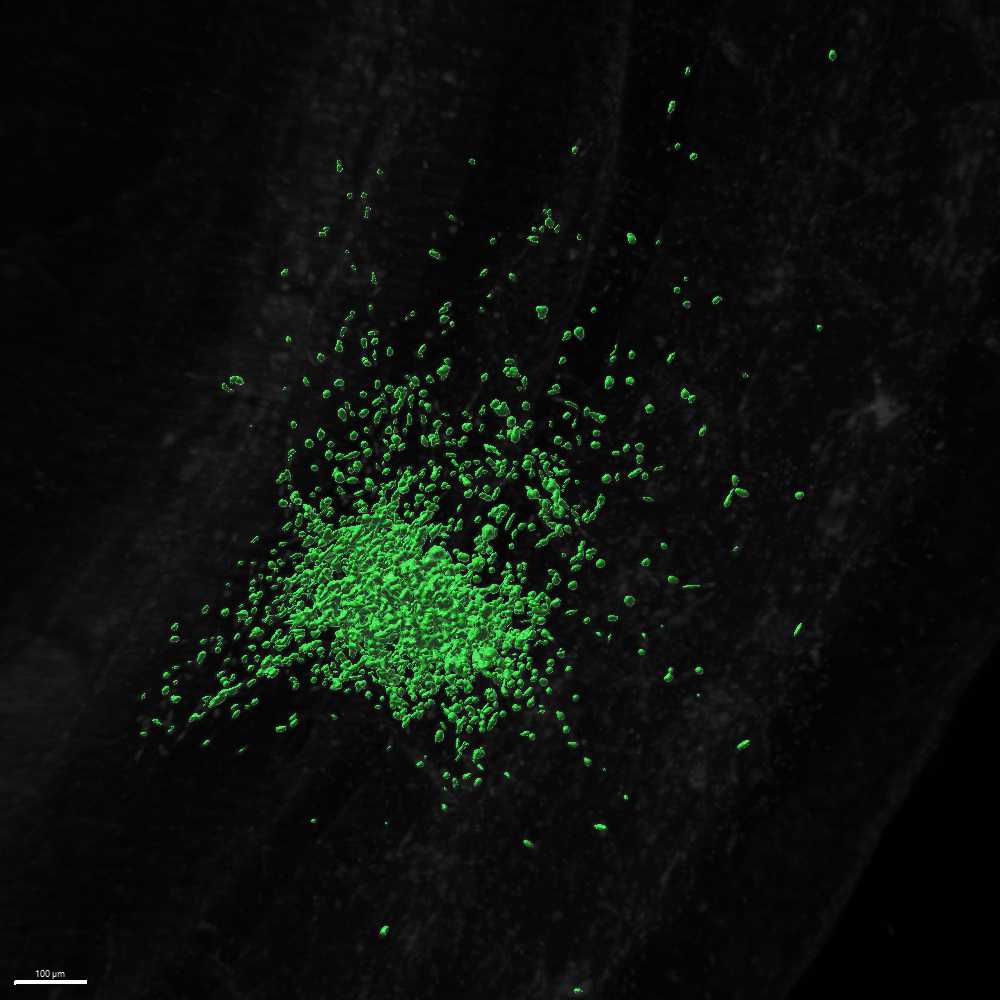

Supplement: Supplementary file 6 — Source data Fig. 5 [file 44321_2026_398_MOESM6_ESM.zip › 2025-21404-Figure5/5B/Embryo - control group- MDAMB 231 cells CFSE volume.jpg]

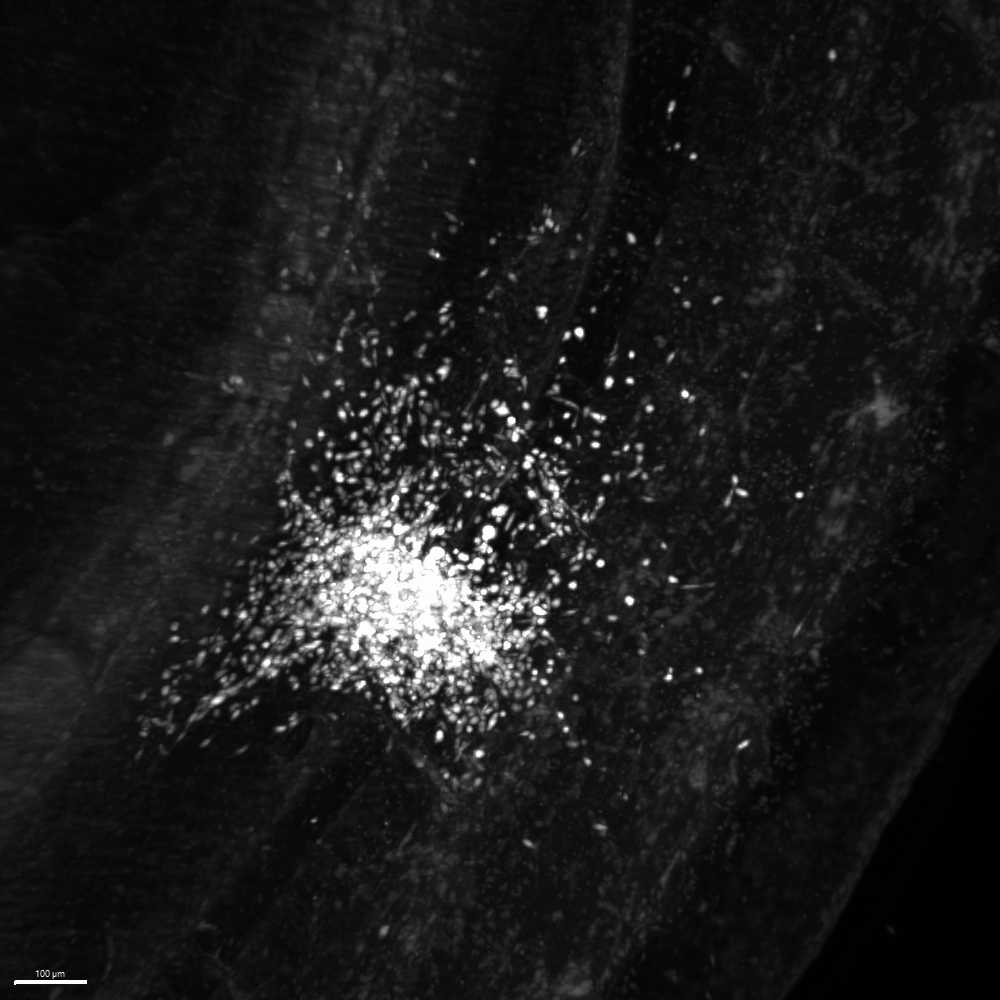

Supplement: Supplementary file 6 — Source data Fig. 5 [file 44321_2026_398_MOESM6_ESM.zip › 2025-21404-Figure5/5B/Embryo - control group- MDAMB 231 cells CFSE.jpg]

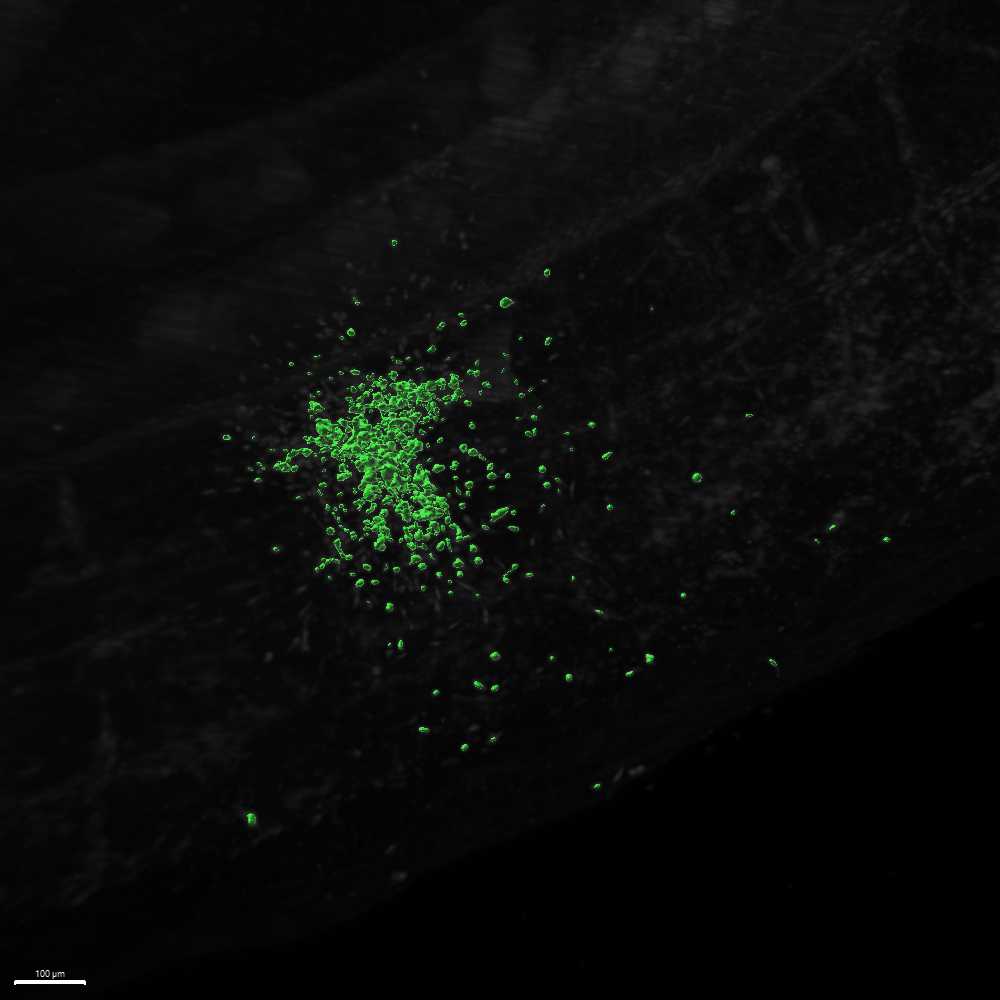

Supplement: Supplementary file 6 — Source data Fig. 5 [file 44321_2026_398_MOESM6_ESM.zip › 2025-21404-Figure5/5B/Embryo - treated group- MDAMB 231 cells CFSE volume.jpg]

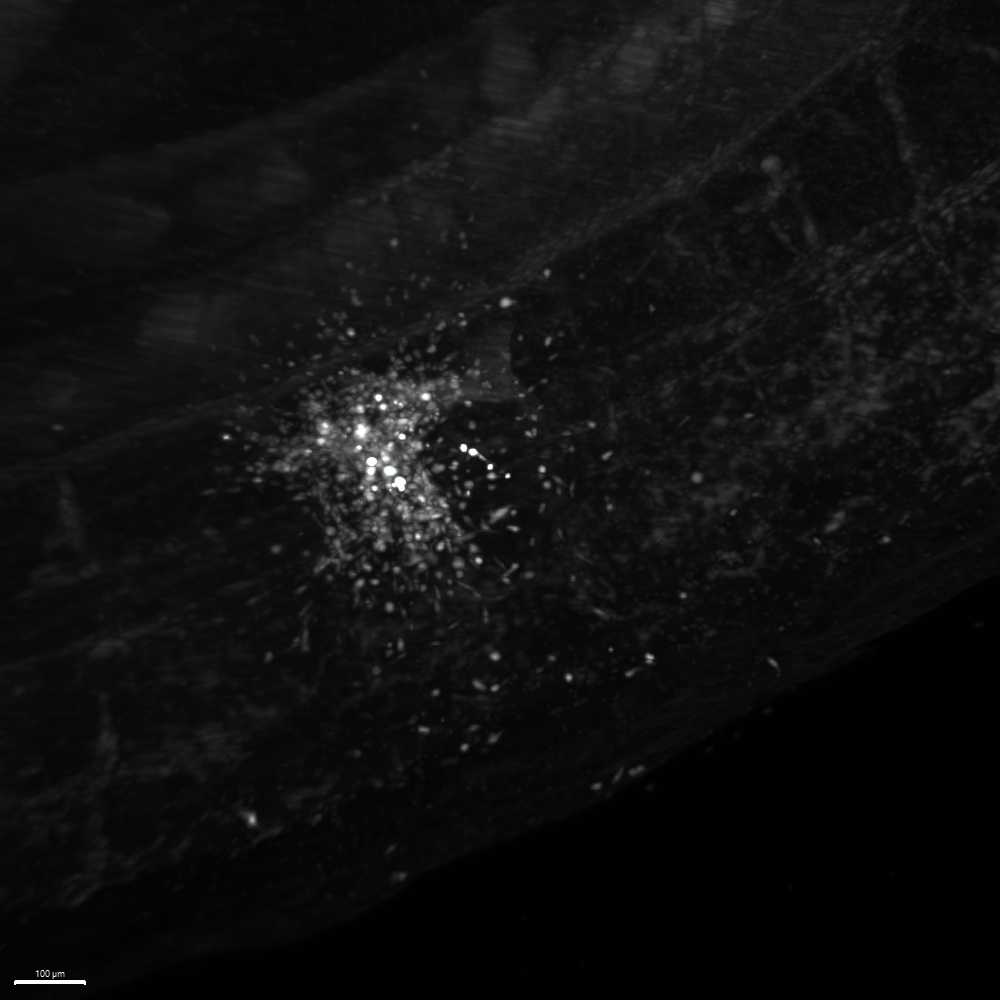

Supplement: Supplementary file 6 — Source data Fig. 5 [file 44321_2026_398_MOESM6_ESM.zip › 2025-21404-Figure5/5B/Embryo - treated group- MDAMB 231 cells CFSE.jpg]

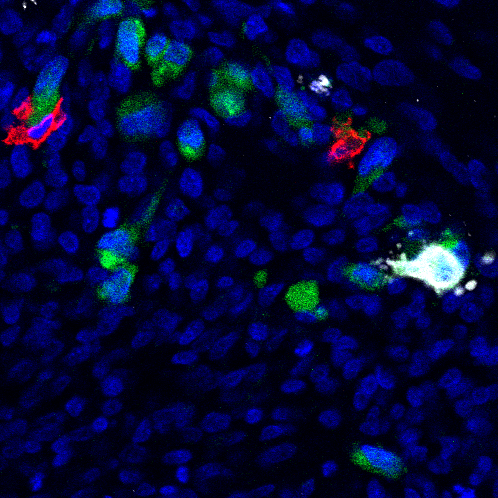

Supplement: Supplementary file 6 — Source data Fig. 5 [file 44321_2026_398_MOESM6_ESM.zip › 2025-21404-Figure5/5G/Section anti-PD-1- blue nucleus- green tumor cells- red Hu-PBMC- white cCaspase3.tif]

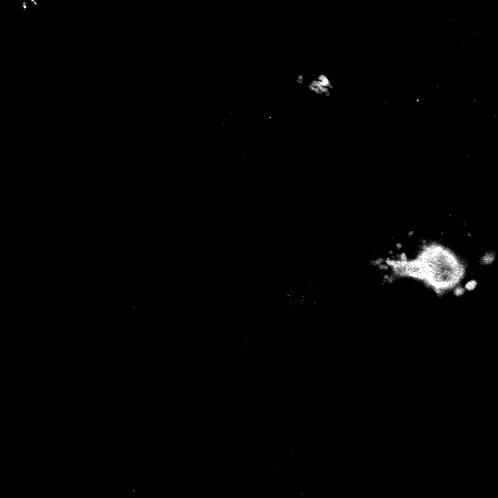

Supplement: Supplementary file 6 — Source data Fig. 5 [file 44321_2026_398_MOESM6_ESM.zip › 2025-21404-Figure5/5G/Section anti-PD-1- white cCaspase3.tif]

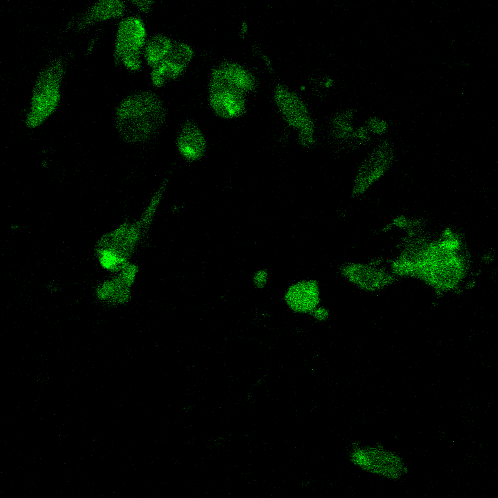

Supplement: Supplementary file 6 — Source data Fig. 5 [file 44321_2026_398_MOESM6_ESM.zip › 2025-21404-Figure5/5G/Section anti-PD-1-green tumor cells.tif]

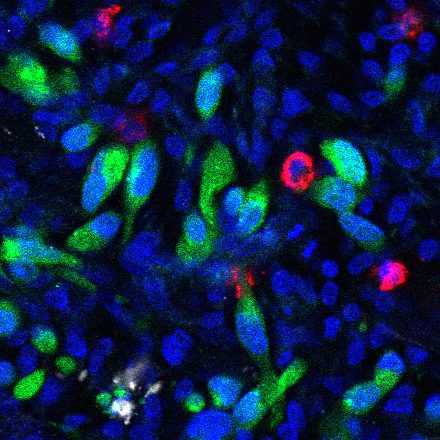

Supplement: Supplementary file 6 — Source data Fig. 5 [file 44321_2026_398_MOESM6_ESM.zip › 2025-21404-Figure5/5G/Section Control- blue nucleus- green tumor cells- red Hu-PBMC- white cCaspase3.tif]

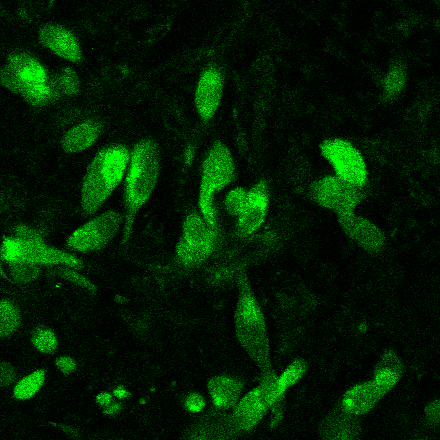

Supplement: Supplementary file 6 — Source data Fig. 5 [file 44321_2026_398_MOESM6_ESM.zip › 2025-21404-Figure5/5G/Section Control- green tumor cells.tif]

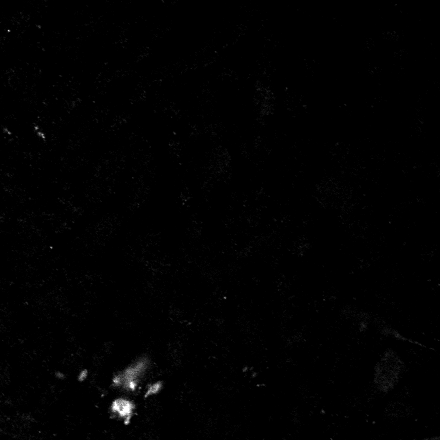

Supplement: Supplementary file 6 — Source data Fig. 5 [file 44321_2026_398_MOESM6_ESM.zip › 2025-21404-Figure5/5G/Section Control- white cCaspase3.tif]

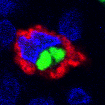

Supplement: Supplementary file 7 — Source data Fig. 6 [file 44321_2026_398_MOESM7_ESM.zip › 2025-21404-Figure6/6A/CRC-03 Hoescht CFSE+tumor 555CD45+-c1 zoom.tif]

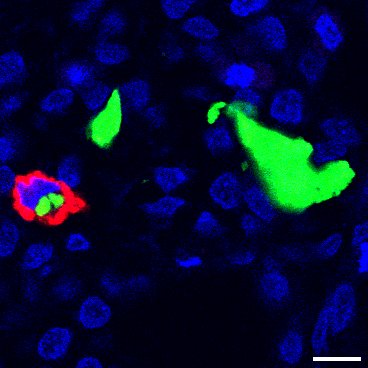

Supplement: Supplementary file 7 — Source data Fig. 6 [file 44321_2026_398_MOESM7_ESM.zip › 2025-21404-Figure6/6A/CRC-03 Hoescht CFSE+tumor 555CD45+-c1.jpg]

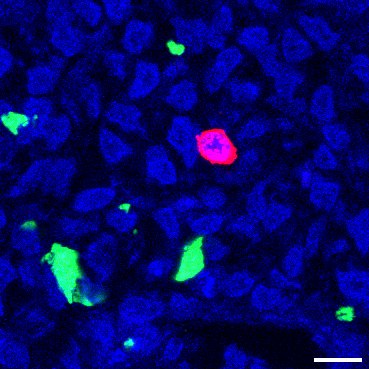

Supplement: Supplementary file 7 — Source data Fig. 6 [file 44321_2026_398_MOESM7_ESM.zip › 2025-21404-Figure6/6A/CRC-03 Hoescht CFSE+tumor 555CD45+-c2.jpg]

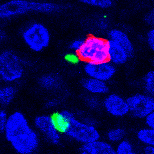

Supplement: Supplementary file 7 — Source data Fig. 6 [file 44321_2026_398_MOESM7_ESM.zip › 2025-21404-Figure6/6A/CRC-03 Hoescht CFSE+tumor 555CD45+-c3.tif]

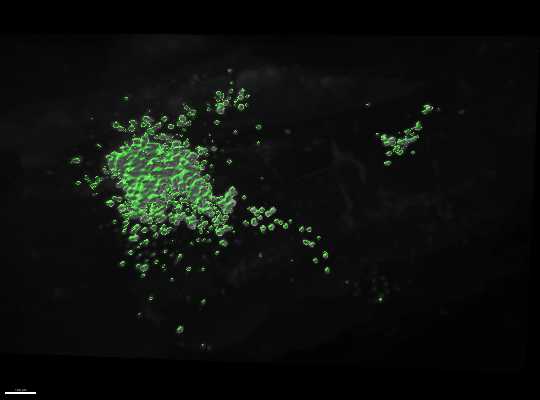

Supplement: Supplementary file 7 — Source data Fig. 6 [file 44321_2026_398_MOESM7_ESM.zip › 2025-21404-Figure6/6B/Embryo- MDAMB 231 CFSE- anti-PD-1 volume.jpg]

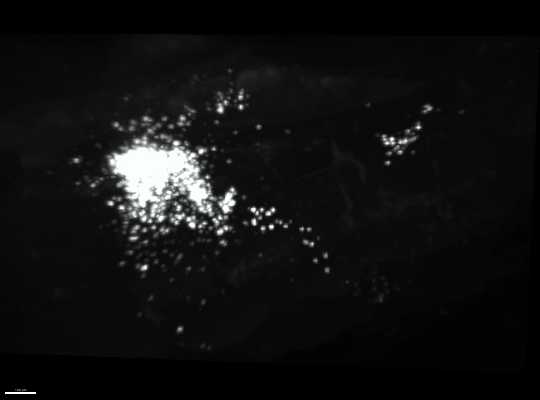

Supplement: Supplementary file 7 — Source data Fig. 6 [file 44321_2026_398_MOESM7_ESM.zip › 2025-21404-Figure6/6B/Embryo- MDAMB 231 CFSE- anti-PD-1.jpg]

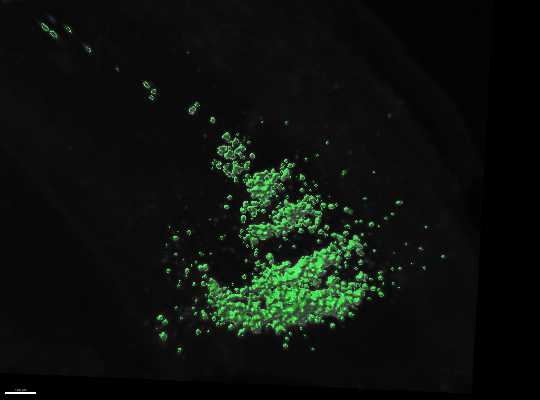

Supplement: Supplementary file 7 — Source data Fig. 6 [file 44321_2026_398_MOESM7_ESM.zip › 2025-21404-Figure6/6B/Embryo- MDAMB 231 CFSE- Control volume.jpg]

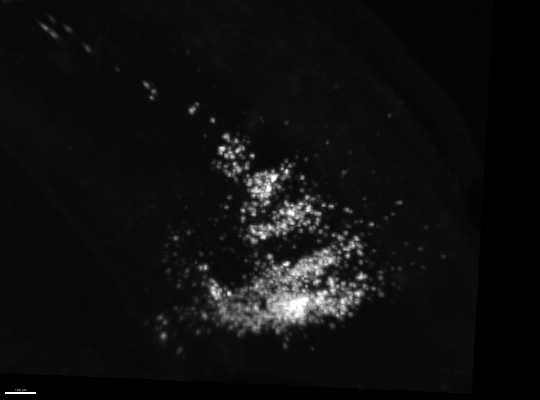

Supplement: Supplementary file 7 — Source data Fig. 6 [file 44321_2026_398_MOESM7_ESM.zip › 2025-21404-Figure6/6B/Embryo- MDAMB 231 CFSE- Control.jpg]

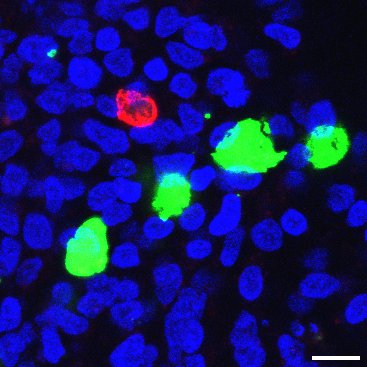

Supplement: Supplementary file 7 — Source data Fig. 6 [file 44321_2026_398_MOESM7_ESM.zip › 2025-21404-Figure6/6G/BRE02- Hoescht CFSE+tumor 555CD45-c1.jpg]

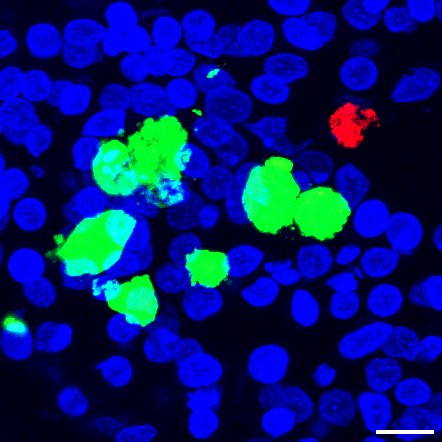

Supplement: Supplementary file 7 — Source data Fig. 6 [file 44321_2026_398_MOESM7_ESM.zip › 2025-21404-Figure6/6G/BRE02- Hoescht CFSE+tumor 555CD45-c2.jpg]

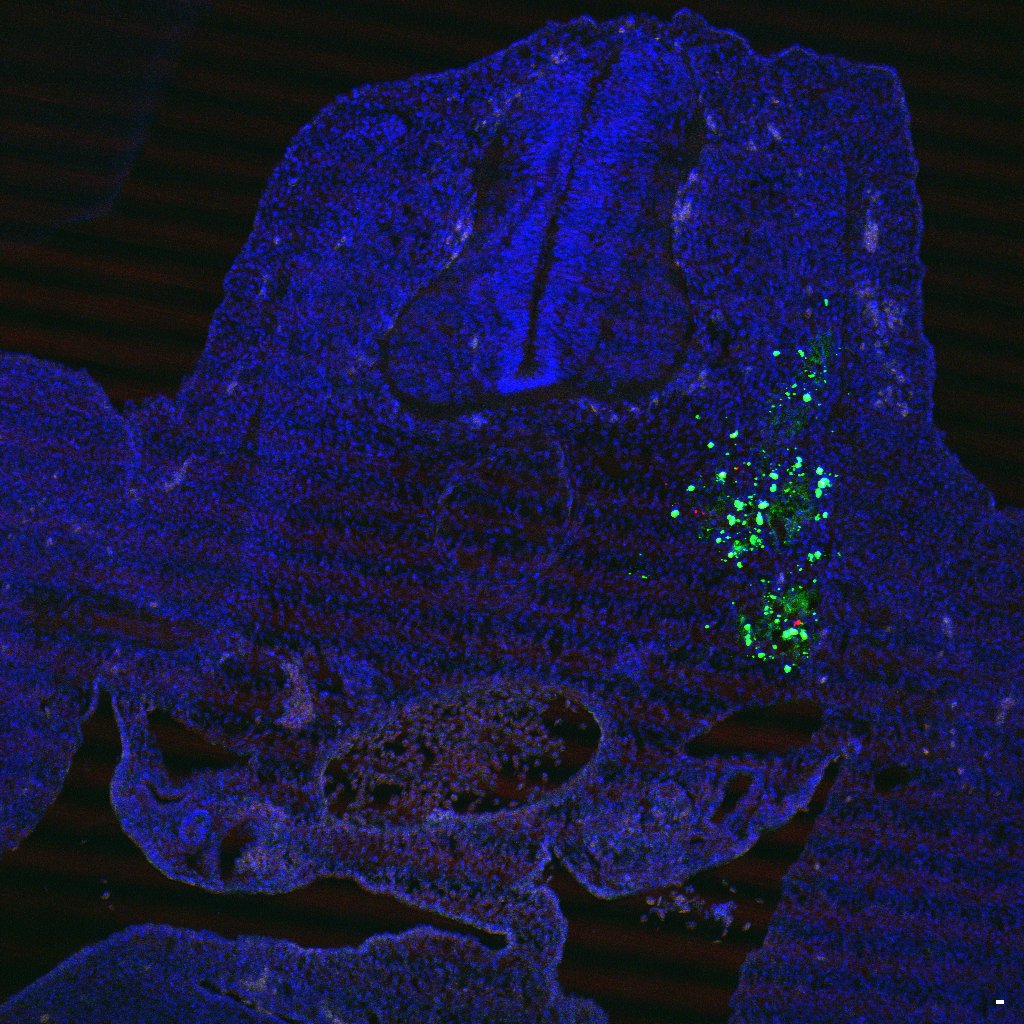

Supplement: Supplementary file 7 — Source data Fig. 6 [file 44321_2026_398_MOESM7_ESM.zip › 2025-21404-Figure6/6G/CRC-07 Hoescht CFSE+tumor OCT hu-PBMC x10.jpg]

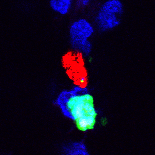

Supplement: Supplementary file 7 — Source data Fig. 6 [file 44321_2026_398_MOESM7_ESM.zip › 2025-21404-Figure6/6G/CRC-07 Hoescht CFSE+tumor OCT hu-PBMC x63 zoom.tif]

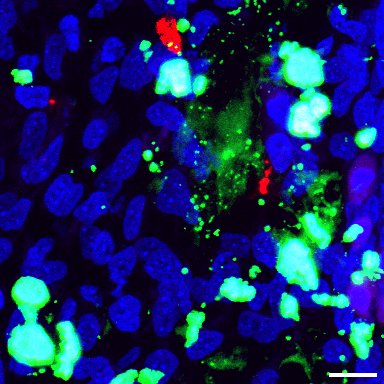

Supplement: Supplementary file 7 — Source data Fig. 6 [file 44321_2026_398_MOESM7_ESM.zip › 2025-21404-Figure6/6G/CRC-07 Hoescht CFSE+tumor OCT hu-PBMC x63-2.jpg]

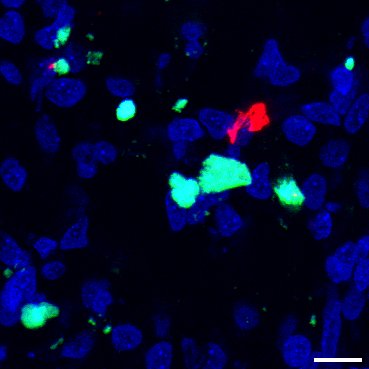

Supplement: Supplementary file 7 — Source data Fig. 6 [file 44321_2026_398_MOESM7_ESM.zip › 2025-21404-Figure6/6G/CRC-07 Hoescht CFSE+tumor OCT hu-PBMC x63.jpg]

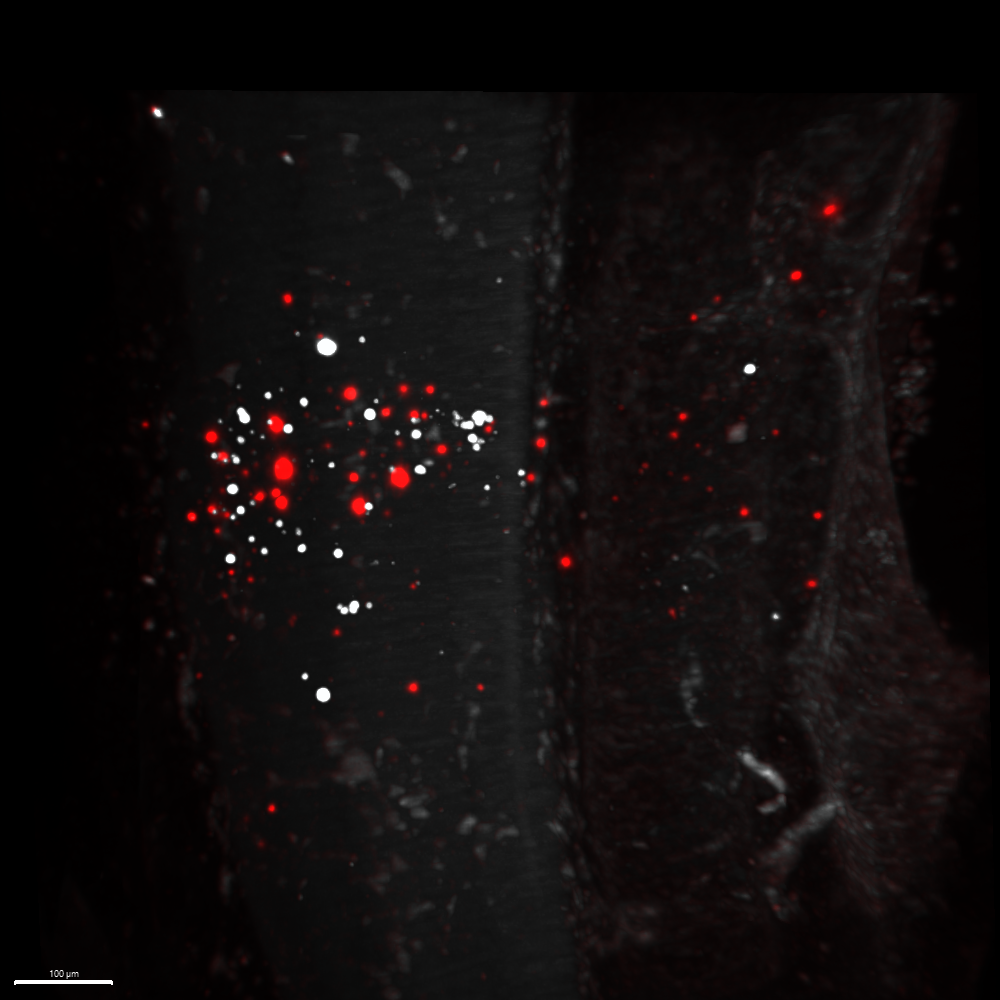

Supplement: Supplementary file 7 — Source data Fig. 6 [file 44321_2026_398_MOESM7_ESM.zip › 2025-21404-Figure6/6H/Embryo-CRC-07+ PBMC autologue-CFSE and OCT .tif]

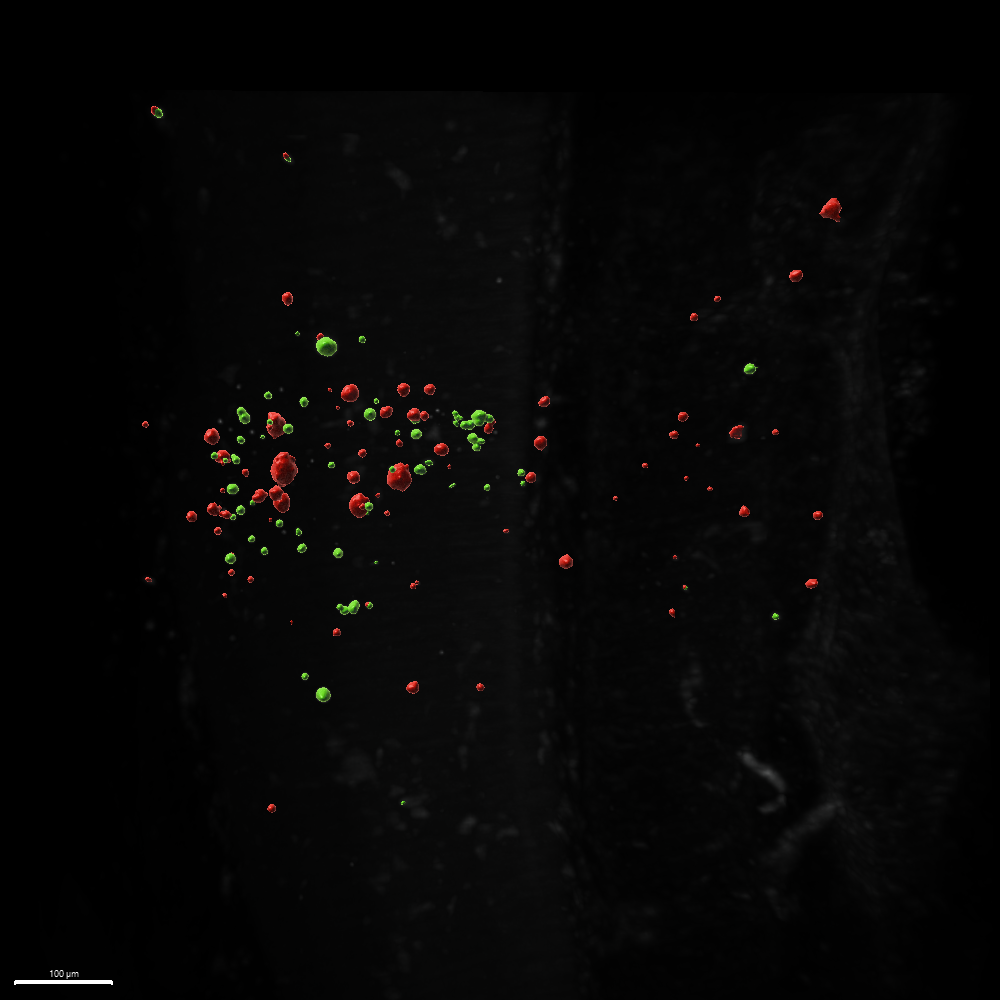

Supplement: Supplementary file 7 — Source data Fig. 6 [file 44321_2026_398_MOESM7_ESM.zip › 2025-21404-Figure6/6H/Embryo-CRC-07+ PBMC autologue-CFSE and OCT volumes.tif]

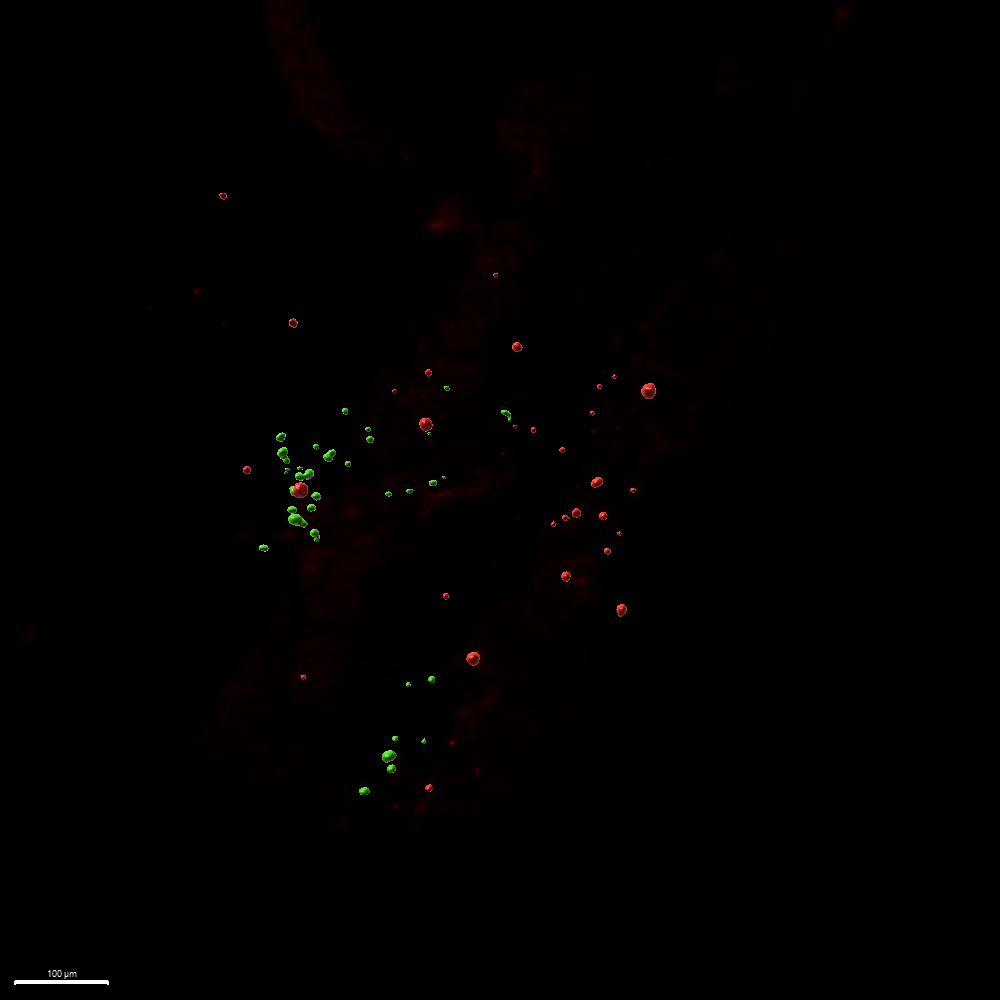

Supplement: Supplementary file 7 — Source data Fig. 6 [file 44321_2026_398_MOESM7_ESM.zip › 2025-21404-Figure6/6H/Embryo-CRC-08+ PBMC autologue-CFSE and OCT volume.tif]

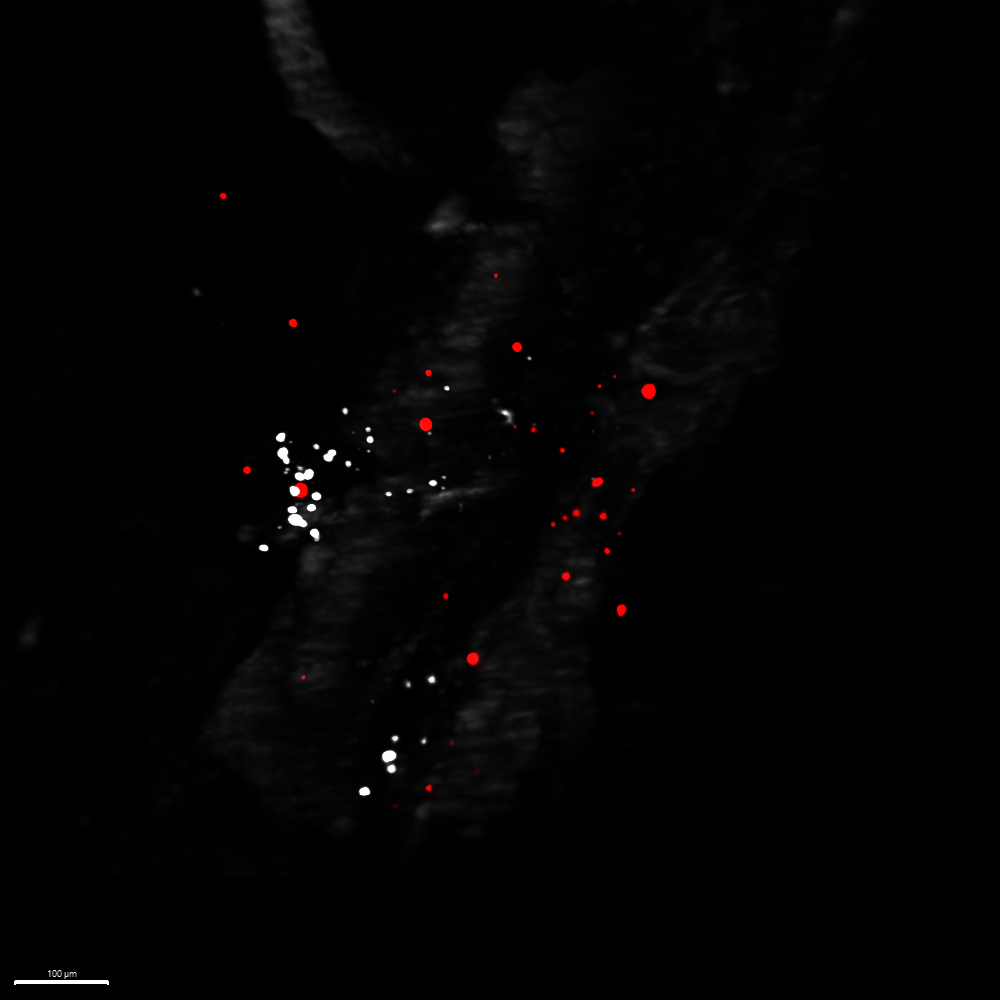

Supplement: Supplementary file 7 — Source data Fig. 6 [file 44321_2026_398_MOESM7_ESM.zip › 2025-21404-Figure6/6H/Embryo-CRC-08+ PBMC autologue-CFSE and OCT.tif]
