# Supplementary material for: Humanized avian embryo models replicate an immune tumor environment for rapid immunotherapy studies
Source: EMBO Mol Med. 2026 Mar 19;18(4):1399–428. doi: 10.1038/s44321-026-00398-5 (PMC13083996; doi:10.1038/s44321-026-00398-5)
Supplement: Supplementary file 3 — Source data Fig. 2 [file 44321_2026_398_MOESM3_ESM.zip › 2025-21404-Figure2/2K/READ ME.docx]

FACS profiles of TIM-3 and PD-1 expressions within the CD3+CD8+ population of hu-PBMCs infiltrated in MDA-MB-231 tumors or control tissues.

The sample analyzed contained both Hu-CD45+ cells and chick embryo cells (majority of cells)

**The gating strategy is :**

FSC/SSC : select only the zone containing immune cells

FSC-A/FSC-H : select single cells

AmCyan : select viable cells (negative cells)

AlexaFluor488 : select negative cells (CFSE+ are tumor cells)

AlexaFluor 405 : select positive cells CD45

BUV395 : CD3+ population. Use low gate to take into account the decrease in CD3 expression of activated cells

7AAD : select positive population CD8

- AlexaFluor 647 : positive cells for PD-1. The gate is placed according the isotype control.
- BV650 : positive cells for TIM-3. The gate is placed according the isotype control.

The FCS files can be found in figshare website following the link : 10.6084/m9.figshare.30647849
