# Supplementary material for: Humanized avian embryo models replicate an immune tumor environment for rapid immunotherapy studies
Source: EMBO Mol Med. 2026 Mar 19;18(4):1399–428. doi: 10.1038/s44321-026-00398-5 (PMC13083996; doi:10.1038/s44321-026-00398-5)
Supplement: Supplementary file 4 — Source data Fig. 3 [file 44321_2026_398_MOESM4_ESM.zip › 2025-21404-Figure3/3C/READ ME.docx]

Representative light sheet microscopy photographs illustrating the quantitative analysis.

Blue circles represent the grafting site

Pink cercle represents the area covered by orange-labeled-hu-PBMCs .
