# Supplementary material for: Humanized avian embryo models replicate an immune tumor environment for rapid immunotherapy studies
Source: EMBO Mol Med. 2026 Mar 19;18(4):1399–428. doi: 10.1038/s44321-026-00398-5 (PMC13083996; doi:10.1038/s44321-026-00398-5)
Supplement: Supplementary file 5 — Source data Fig. 4 [file 44321_2026_398_MOESM5_ESM.zip › 2025-21404-Figure4/4F/READ ME.docx]

FACS profiles of CD69 and CD25 expressions within the CD3+CD8+ population of hu-PBMCs co-grafted with MDA-MB-231 cells in embryos treated either with NaCl or with pembrolizumab.

The analyzed samples contain both Hu-CD45+ cells and chick embryo cells (majority of cells)

**The gating strategy is :**

FSC/SSC : select only the zone containing immune cells

FSC-A/FSC-H : select single cells

V510 : select viable cells (negative cells)

CFSE : select negative cells (CFSE+ are tumor cells)

V450 : select positive cells CD45

BUV395 : CD3+ population. Use low gate to take into account the decrease in CD3 expression of activated cells

PerCPCy 5.5 : select positive population CD8

PI : positive cells for CD69. The gate is placed according to the isotype control.

BUV 737 : positive cells for CD25. The gate is placed according to the isotype control.

The FCS files can be found in figshare website following the link : 10.6084/m9.figshare.30647879
