# Supplementary material for: Humanized avian embryo models replicate an immune tumor environment for rapid immunotherapy studies
Source: EMBO Mol Med. 2026 Mar 19;18(4):1399–428. doi: 10.1038/s44321-026-00398-5 (PMC13083996; doi:10.1038/s44321-026-00398-5)
Supplement: Supplementary file 7 — Source data Fig. 6 [file 44321_2026_398_MOESM7_ESM.zip › 2025-21404-Figure6/6D/READ ME.docx]

FACS profiles of PD-L1 expression in indicated colorectal samples : CRC-01, CRC-02, CRC-03, CRC-04, CRC-05, CRC-06.

For each colorectal sample, the IgG sample is used for placing the PD-L1 gate.

**The gating strategy is :**

FSC/SSC : select cell population

FSC-A/FSC-H : select single cells

APC/FSC-A : expression of PD-L1.
