# Supplementary material for: Humanized avian embryo models replicate an immune tumor environment for rapid immunotherapy studies
Source: EMBO Mol Med. 2026 Mar 19;18(4):1399–428. doi: 10.1038/s44321-026-00398-5 (PMC13083996; doi:10.1038/s44321-026-00398-5)
Supplement: Supplementary file 8 — Expanded View Figures [file 44321_2026_398_MOESM8_ESM.pdf]

Expanded View Figures

Figure EV1. The paradigms of avian humanization create a tumor microenvironment.

(**A**) Representative FACS profiles of CD69 and CD25 activation markers or TIM-3 and PD-1 exhaustion marker expressions within the CD3+ CD4+ population of hu-PBMCs infiltrated in MDA-MB-231 tumor site or in equivalent control tissues. Data are presented for 37 pooled embryos intra-venously injected with hu-PBMCs from donor 5. (**B**) Histograms showing the quantification of tumor volumes in embryos grafted with CFSE+ MDA-MB-231 cell line, and intra-venously injected or not with hu-PBMCs from donor 4. $N = 1$ experiment ($n = 5$ embryos in control group and $n = 8$ embryos in I.V injected group). Dots represent volumes normalized to each embryo's BSA. Data are represented as mean ± SEM. Mann–Whitney test, exact *P*-values indicated on graph. ns: not significant. (**C**) Histogram showing the fraction of Caspase 3 positive cells within the avian somitic tissue, in presence and absence of hu-PBMCs-MDA-MB-231 cells (hu-PBMCs from donor 6). For each experimental group, $n = 7$ sections from 3 embryos. Mann–Whitney test, exact *P*-values indicated on the graph. ns: not significant. (**D**) Microphotographs of immunofluorescent labeling of cryosections of chick embryos at 48 h post co-grafting of CFSE+ MDA-MB-231 cells (green) and hu-PBMCs from donor 6. Immune cells were detected with anti-human CD45 antibody (red), and apoptotic cells with anti-cleaved Caspase 3 antibody (white). Nuclei of avian and human cells were stained with Hoechst (blue). The images show somitic tissues free or populated by grafted human cells. White arrow indicates avian Caspase 3+ cells. (**E**) Histogram showing the global distribution pattern of hu-PBMCs from donor 10 when grafted alone ($n = 9$ embryos) or in combination with MDA-MB-231 cells at a ratio 1:1 ($n = 11$ embryos), $N = 1$. Data are represented as mean ± SEM. Each dot represents the average distance of hu-PBMCs from the site of injection for individual embryos. Mann–Whitney test, exact *P*-value indicated on graph. $^{**}P < 0.01$. (**F**, **G**) Histograms showing the differences of hu-PBMC subpopulation fractions for two donors, between conditions of pre-grafting, grafting alone or grafting in combination with MDA-MB-231 or HCT 116 cells. Embryos were harvested 48 h post grafting. $N = 3$ for pre- and post-graft groups. For the histogram in (**F**), $N = 3$ for co-grafted experimental group ($n = 8$ to 11 pooled embryos); For the histogram in (**G**), $N = 2$ for co-engrafted experimental group ($n = 7$ to 9). The histogram of post-graft is also presented in Fig. 1G. Data are represented as mean ± SEM. Chi-square test, *P*-values are indicated. $^{****}P < 0.0001$, $^{***}P < 0.001$ ($P = 0.0003$), $^{*}P < 0.05$ ($P = 0.0373$). (**H**, **I**) Histograms showing the percentage of CD69 positive cells (**H**) and TIM-3 positive cells (**I**) within the CD3+ CD8+ and CD3+ CD4+ populations, for 3 donors in two experimental groups of hu-PBMCs grafted alone or co-grafted with MDA-MB-231 cells. Donors are color-coded (red: donor 1, orange: donor 6 and blue: donor 8). Each dot represents the result of pooled embryos, see details Fig. 3H,I. Mann–Whitney test, *P*-values are indicated on the graph. $^{*}P < 0.05$, ns: not significant.

 © The Author(s)

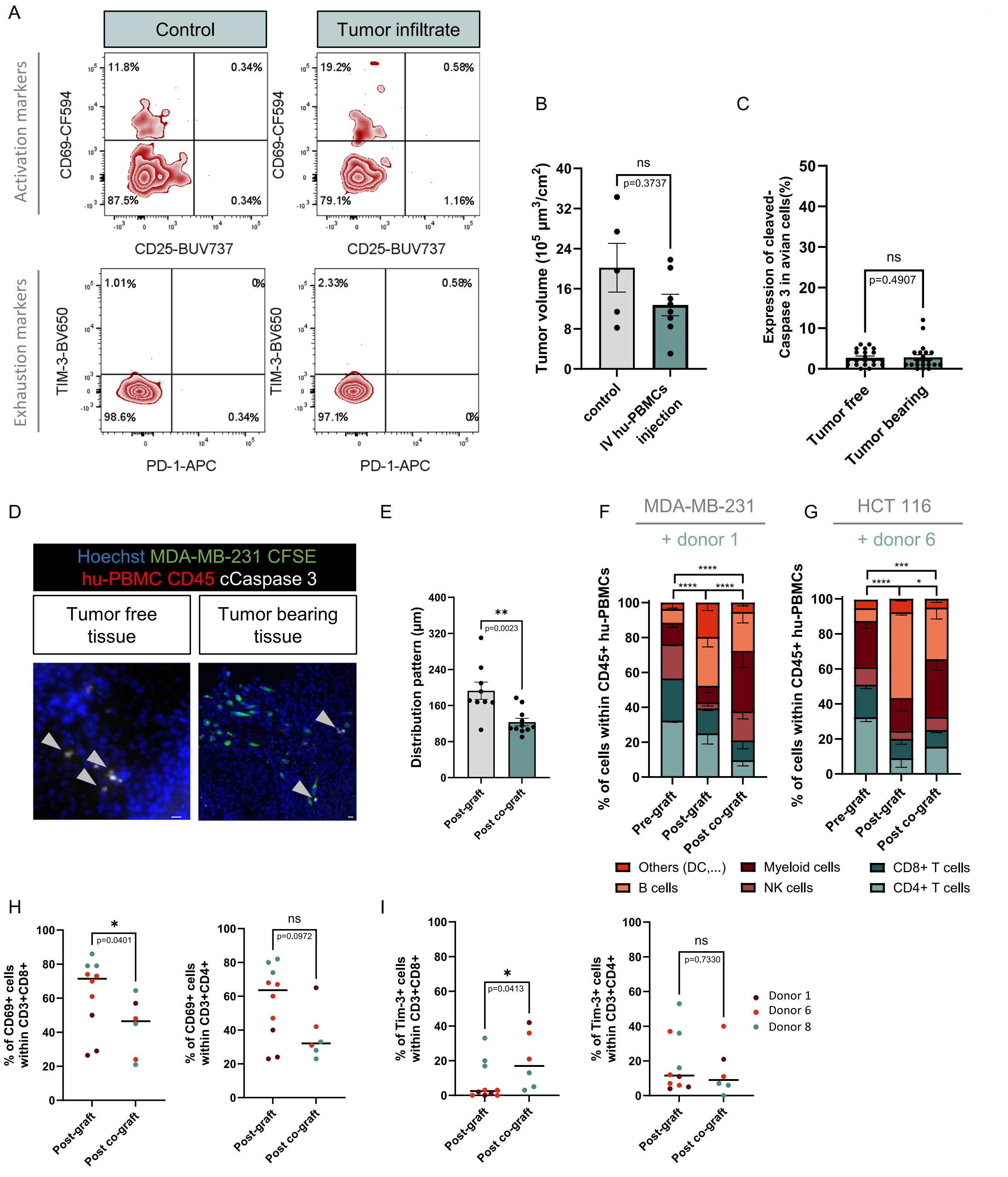
A
Control
Tumor infiltrate
Activation markers
CD69-CF594
CD25-BUV737
11.8%
0.34%
87.5%
0.34%
19.2%
0.58%
79.1%
1.16%
Exhaustion markers
TIM-3-BV650
PD-1-APC
1.01%
0%
98.6%
0.34%
2.33%
0.58%
97.1%
0%
B
Tumor volume (10^5 µm^3/cm^2)
ns
p=0.3737
control
IV hu-PBMCs injection
C
Expression of cleaved-Caspase 3 in avian cells(%)
ns
p=0.4907
Tumor free
Tumor bearing
D
Hoechst MDA-MB-231 CFSE
hu-PBMC CD45 cCaspase 3
Tumor free tissue
Tumor bearing tissue
E
Distribution pattern (µm)
**
p=0.0023
Post-graft
Post co-graft
F
MDA-MB-231
+ donor 1
% of cells within CD45+ hu-PBMCs
Pre-graft
Post-graft
Post co-graft
G
HCT 116
+ donor 6
Others (DC,...)
B cells
Myeloid cells
NK cells
CD8+ T cells
CD4+ T cells
H
% of CD69+ cells within CD3+CD8+
*
p=0.0401
% of CD69+ cells within CD3+CD4+
ns
p=0.0972
I
% of Tim-3+ cells within CD3+CD8+
*
p=0.0413
% of Tim-3+ cells within CD3+CD4+
ns
p=0.7330
Donor 1
Donor 6
Donor 8

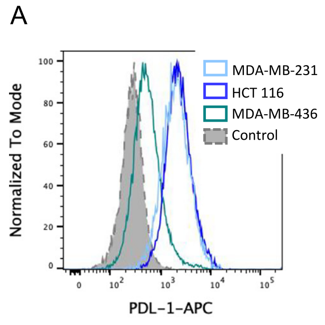

B

Pembrolizumab dose (mg/kg)	Number of abnormal embryos/ 15 embryos				
	Craniofacial morphology	Eye morphology	4 limb buds	Cardiac morphology	Embryonic annexes
Excipient	0	0	0	0	0
35.6	0	0	0	0	0
178	0	0	0	0	0
890	1	1	0	0	0

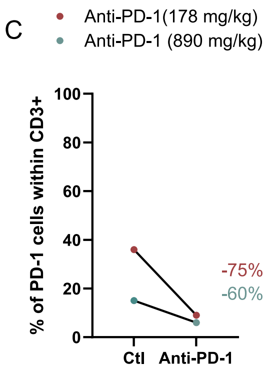

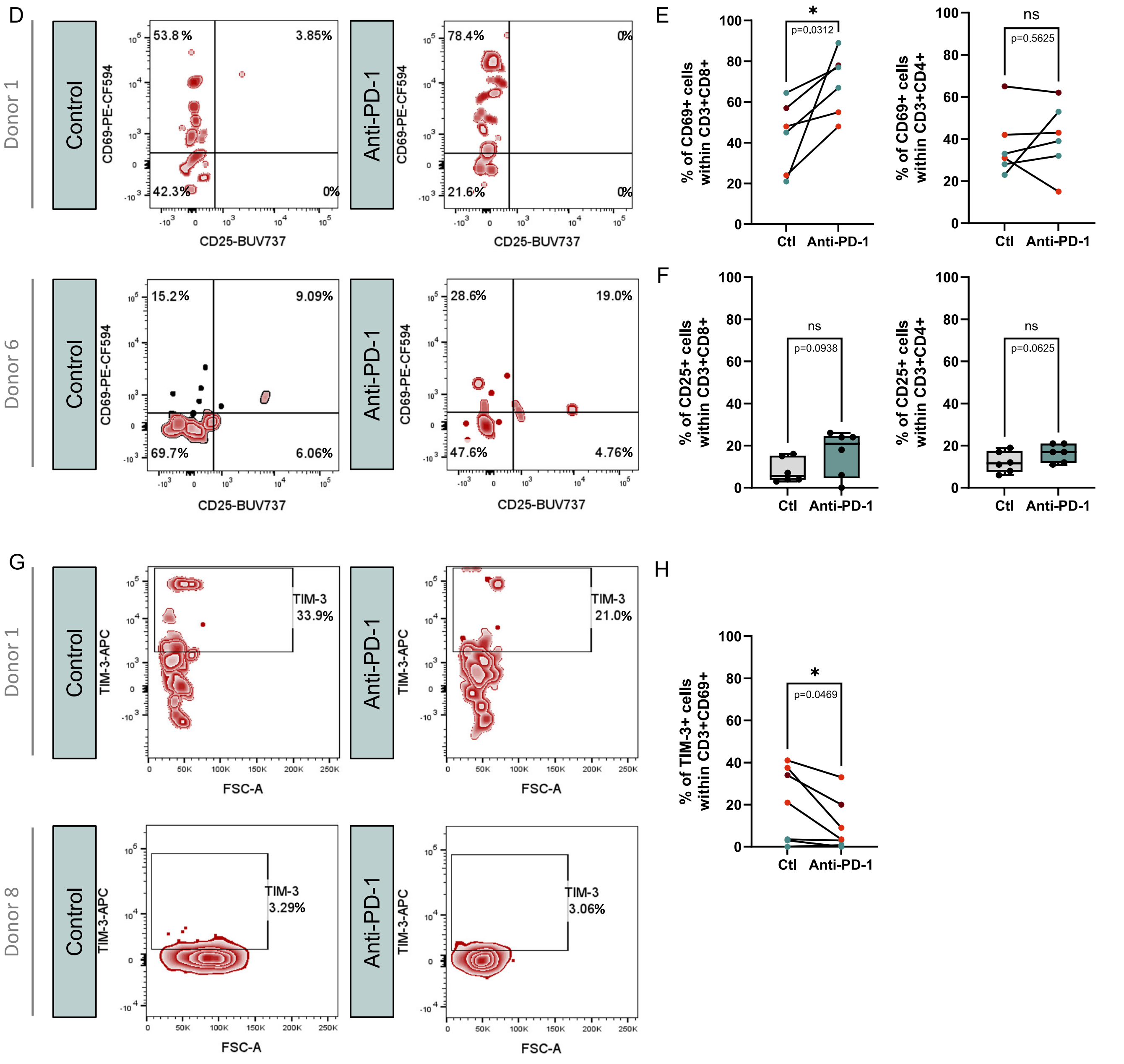

© The Author(s)

Figure EV2. The anti-PD-1 administration impacts human PBMCs.

(**A**) Representative FACS profiles of PD-L1 expression in indicated cell lines (MDA-MB-231, MDA-MB-436, HCT 116). (**B**) Table presenting the morphological analysis of embryos treated with different pembrolizumab doses. (**C**) Histogram showing the fraction of PD-1 positive cells in CD3+ populations in embryos grafted with a mix of hu-PBMCs from donor 1 and MDA-MB-231 cells, treated either with anti-PD-1 (pembrolizumab) at 2 doses (178 in red or 890 mg/kg in blue) or control (NaCl 0.9%). Percentages represent the PD-1 expression diminution in pembrolizumab condition compared to control. $N = 1$, for 178 mg/kg dose ($n = 51$ pooled embryos in control group; $n = 54$ pooled embryos in pembrolizumab group) also shown in Fig. 4D; for 890 mg/kg ($n = 29$ and $n = 36$). No statistical test was performed. (**D**) Representative FACs profiles of CD69 and CD25 expression in the CD3+ CD8+ population of hu-PBMCs in embryos grafted with hu-PBMCs from donors 1 or 6, combined with MDA-MB-231 cells and treated either with control (NaCl 0.9%) or anti-PD-1 (pembrolizumab). Donor 1 ($n = 52$ pooled embryos per experimental group); donor 6 ($n = 20$ in control group; $n = 26$ in pembrolizumab group). (**E**) Histogram showing the fraction of CD69 positive cells in CD3+ CD8+ and CD3+ CD4+ populations in embryos grafted with a mix of hu-PBMCs and MDA-MB-231 cells and treated either with anti-PD-1 (pembrolizumab) or control (NaCl 0.9%). Each dot represents pooled embryos in experiments conducted with 3 donors (red: donors 1, orange: donor 6; blue: donor 8), see details Fig. 4E. Wilcoxon test, exact *P*-values indicated on the graph. *$P < 0.05$, ns: not significant. (**F**) Histograms showing the fraction of CD25 positive cells in the CD3+ CD8+ and CD3+ CD4+ populations in hu-PBMCs co-grafted with MDA-MB-231 cells in embryos treated either with anti-PD-1 (pembrolizumab) or with control (NaCl 0.9%). Each dot represents pooled embryos in experiments conducted with 3 donors (donors 1, 6 and 8). $N = 1$ for donor 1 ($n = 51$ pooled embryos in control group; $n = 54$ pooled embryos in pembrolizumab group); $N = 2$ donor 6 ($n = 17$ to 33; $n = 27$ to 36); $N = 3$ donor 8 ($n = 24$ to 27; $n = 19$ to 23). Experiments with fewer than 20 CD3+ CD8+ or 20 CD3+ CD4+ cells were excluded from analysis. Box-plots represent the median, interquartile range (25th–75th percentiles), with whiskers indicating the minimum and maximum values. Wilcoxon test, exact *P*-values indicated on the graph, ns: not significant. (**G**) Representative FACS profiles of TIM-3 expression in the CD3+ CD69+ population in embryos grafted with hu-PBMCs from donor 1 or 8, combined with MDA-MB-231 cells and treated either with control (NaCl 0.9%) or anti-PD-1 (pembrolizumab). Donor 1 ($n = 52$ pooled embryos per experimental group); donor 8 ($n = 24$ in control group; $n = 19$ in pembrolizumab group). (**H**) Histogram showing the fraction of TIM-3 positive cells in CD3+ CD69+ population in embryos grafted with a mix of hu-PBMCs and MDA-MB-231 cells and treated either with anti-PD-1 (pembrolizumab) or control (NaCl 0.9%). Each dot represents pooled embryos in experiments conducted with 3 donors (red: donors 1, orange: donor 6; blue: donor 8), see details Fig. 4G. Wilcoxon test, exact *P*-value indicated on the graph. *$P < 0.05$.

© The Author(s)

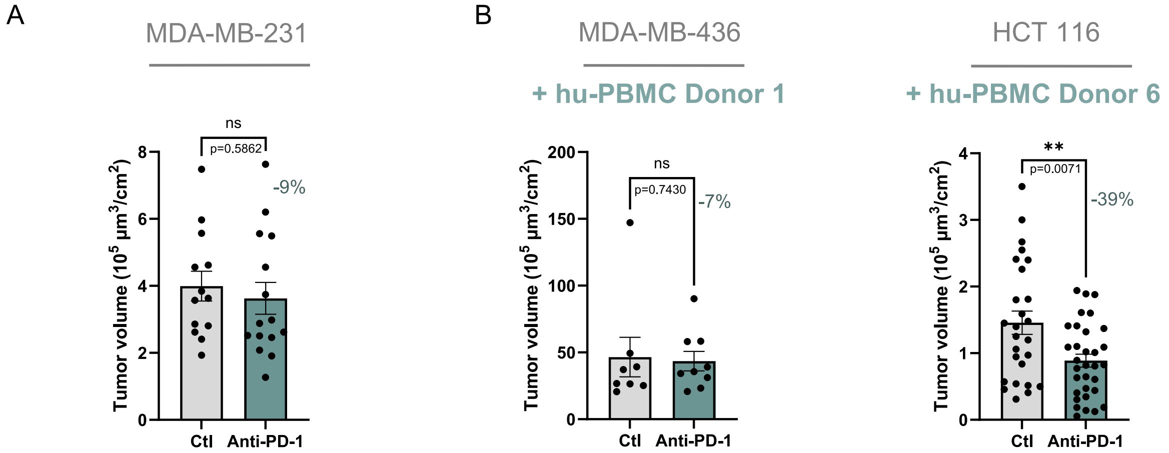

C

Samples	Hu-PBMCs from healthy donors										Cell lines		
Donor number and cell line	1	2	3	4	5	6	7	8	9	10	MDA-MB-231	MDA-MB-436	HCT 116
HLA-A2 haplotype	+	-	undetermined	-	-	-	+	+	+	-	+	-	+

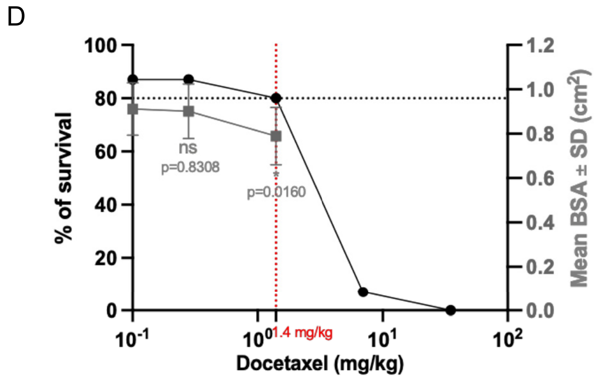

Figure EV3. The anti-PD1 anti-tumoral effect is PBMC donor-dependent.

(**A**) Histograms showing the quantification of the tumor volumes of MDA-MB-231, treated either with control (NaCl 0.9%) or anti-PD-1 (pembrolizumab). Dots represent volumes normalized to each embryo's BSA. $N = 1$ ($n = 13$ in control group and $n = 15$ in anti-PD-1 group). Data are represented as mean ± SEM. The percentage represents the average volume reduction. Unpaired T test, exact *P*-value indicated on graph, ns: not significant. (**B**) Histograms showing the quantification of the tumor volumes of MDA-MB-436 cells, and HCT 116 cells co-engrafted with hu-PBMCs from donor 1 or 6, respectively. Embryos were treated either with control (NaCl 0.9%) or anti-PD-1 (pembrolizumab). Dots represent volumes normalized to each embryo's BSA. $N = 1$ experiment per cell line, for MDA-MB-436 ($n = 8$ in control group and $n = 9$ in anti-PD-1 group); for HCT 116 ($n = 26$ and $n = 32$). Data are represented as mean ± SEM. The percentage represents the average volume reduction. Statistical analyses were conducted with Mann–Whitney for MDA-MB-436 and T test with Welch's correction for HCT 116. Exact *P*-values indicated on graphs. $^{**}P < 0.01$, ns: not significant. (**C**) Table showing the characterization of HLA-A2 haplotypes of the different hu-PBMC donors and cancer cell lines. (**D**) Analysis of survival rate (left axis) and mean body surface area (BSA, right axis) of chick embryos injected with increasing doses of docetaxel (0.28, 1.4, 7, 35 mg/kg). Each dose and control (NaCl 0.9%) were injected to 15 embryos, $N = 1$. The dose 1.4 mg/kg was chosen as MTD. Data are represented as mean ± SD. Unpaired T-test compared to excipient, exact *P*-values indicated on the graph, ns: non-significative.

 © The Author(s)

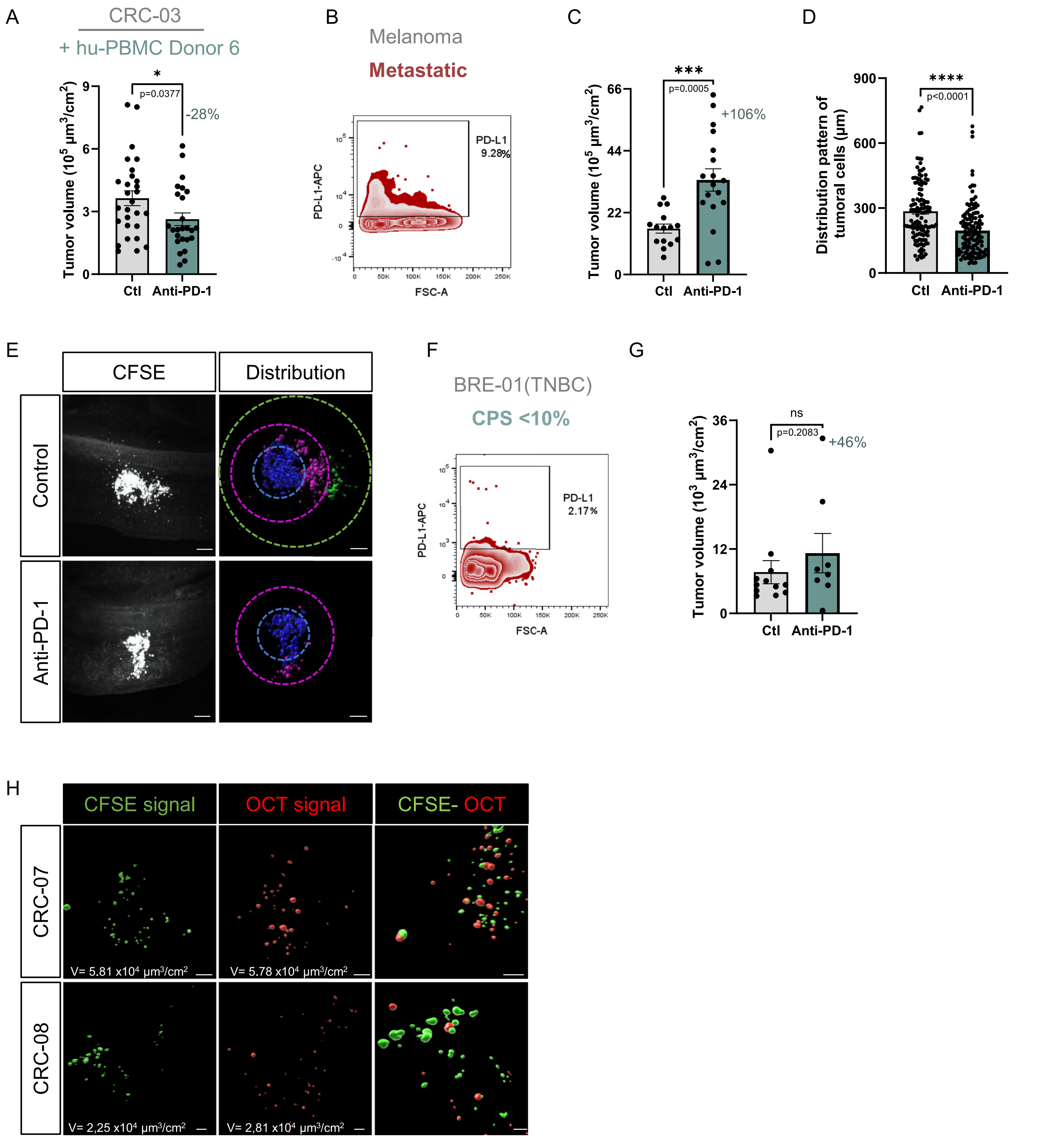

A
CRC-03
+ hu-PBMC Donor 6
Tumor volume (10^5 µm^3/cm^2)
p=0.0377
-28%
Ctl
Anti-PD-1
B
Melanoma
Metastatic
PD-L1
9.28%
PD-L1-APC
FSC-A
C
p=0.0005
+106%
D
Distribution pattern of tumoral cells (µm)
p<0.0001
E
CFSE
Distribution
Control
Anti-PD-1
F
BRE-01(TNBC)
CPS <10%
PD-L1
2.17%
G
ns
p=0.2083
+46%
Tumor volume (10^3 µm^3/cm^2)
H
CFSE signal
OCT signal
CFSE- OCT
CRC-07
CRC-08
V= 5.81 x10^4 µm^3/cm^2
V= 5.78 x10^4 µm^3/cm^2
V= 2,25 x10^4 µm^3/cm^2
V= 2,81 x10^4 µm^3/cm^2

Figure EV4. The anti-PD-1 exerts an effect on several types of patient tumors.

(**A**) Histograms showing the quantification of tumor volumes in embryos co-grafted with CRC-03 patient cells and hu-PBMCs from donor 6, treated with anti-PD-1 (pembrolizumab) or control (NaCl 0.9%). Dots represent volumes normalized to each embryo's BSA. $N = 1$ ($n = 28$ embryos in control group and $n = 25$ embryos in pembrolizumab group). Data are represented as mean ± SEM. The percentage represents the average volume reduction compared to control. Unpaired T test, exact *P*-value indicated on the graph. *$P < 0.05$. (**B**) Representative FACs profile of PD-L1 expression in a patient metastasis melanoma sample. (**C**) Histograms depicting the tumor volumes in embryos co-grafted with metastatic melanoma patient cells and hu-PBMCs from donor 1 and treated with anti-PD-1 (pembrolizumab) or control (NaCl 0.9%). Dots represent volumes normalized to each embryo's BSA. $N = 1$ ($n = 15$ embryos in control group and $n = 18$ in pembrolizumab group). Data are represented as mean ± SEM. The percentage represents the average volume reduction compared to control. Unpaired T test, exact *P*-value indicated on the graph. ***$P < 0.001$. (**D**) Histogram showing the average spreading distances calculated for the 10 cells furthest from the injection site of metastatic melanoma tumor per embryo. Dots represent individual embryos. $N = 1$ ($n = 15$ in control group and $n = 18$ in pembrolizumab group). Data are represented as mean ± SEM. Unpaired T test, exact *P*-value indicated on the graph. ****$P < 0.0001$. (**E**) Microphotographs of CFSE-labeled tumors imaged in light sheet microscopy and segmentation of spreading profiles using Imaris software. Images were taken from 2 representative E4 chick embryos co-grafted with metastatic melanoma patient sample and hu-PBMCs from donor 1, then treated with anti-PD-1 (pembrolizumab) or control (NaCl 0.9%). Spreading distances are segmented in 3 color coded circles: d < 150 µm in blue, 150 µm < d < 300 µm in pink, d > 300 µm in green. Scale bars: 150 µm. (**F**) Representative FACS profiles of PD-L1 expression in a triple-negative breast cancer (TNBC) patient sample (BRE-01) and its combined positive score (CPS). (**G**) Histogram showing the quantification of volume of tumors formed in embryos co-grafted with TNBC patient (BRE-01) cells and hu-PBMCs from donor 1 in embryos treated with anti-PD-1 (pembrolizumab) or control (NaCl 0.9%). Dots represent volumes normalized to each embryo's BSA. $N = 1$ ($n = 10$ embryos in control group and $n = 6$ in pembrolizumab group). Data are represented as mean ± SEM. The percentage represents the average volume reduction compared to control. Mann–Whitney test, exact *P*-value indicated on the graph. ns: not significant. (**H**) Representative light sheet microscopy photographs illustrating CFSE+ tumors from colorectal patient samples (CRC-07 and CRC-08) co-engrafted with their Orange Cell Tracker+ autologous hu-PMBCs, in E4 chick embryos. Panels show the normalized volume (V) for CFSE (in green) and for OCT (in red) fluorescent signals obtained with Imaris software. Scale bars: 50 µm.

 © The Author(s)
